# A Novel 3α-*p*-Nitrobenzoylmultiflora-7:9(11)-diene-29-benzoate and Two New Triterpenoids from the Seeds of Zucchini (*Cucurbita pepo* L)

**DOI:** 10.3390/molecules18077448

**Published:** 2013-06-26

**Authors:** Reiko Tanaka, Takashi Kikuchi, Saori Nakasuji, Yasuhiro Ue, Daisuke Shuto, Keishi Igarashi, Rina Okada, Takeshi Yamada

**Affiliations:** Laboratory of Medicinal Chemistry, Osaka University of Pharmaceutical Sciences, 4-20-1 Nasahara, Takatsuki, Osaka 569-1094, Japan

**Keywords:** *Cucurbita pepo* L, zucchini, 3α-*p*-nitrobenzoylmultiflora-7:9(11)-dien-29-benzoate, 3α-acetoxymultiflora-7:9(11)-diene-29-benzoate, 3α-acetoxymultiflora-5(6):7:9 (11)-triene-29-benzoate

## Abstract

Three novel multiflorane-type triterpenoids, 3α-*p*-nitrobenzoylmultiflora-7:9(11)-diene-29-benzoate (**1**), 3α-acetoxymultiflora-7:9(11)-diene-29-benzoate (**2**), and 3α-acetoxymultiflora-5(6):7:9(11)-triene-29-benzoate (**3**), along with two known related compounds **4** and **5** were isolated from the seeds of zucchini (*Cucurbita pepo* L). Their structures were determined on the basis of 1D and 2D NMR spectroscopy and HREIMS. Triterpenoids possessing a nitro group were not isolated previously.

## 1. Introduction

The species *Cucurbita pepo* is a cultivated plant of the genus *Cucurbita* that includes varieties of squash, gourd, and pumpkin.* Cucurbita pepo* L (zucchini, also known as field pumpkin or summer squash) (Cucurbitaceae) are widely cultivated in America, Europe, and Asia. The zucchini is a hybrid of the cucumber, and has been a commercially important crop in many countries since the 1950–1960s. It is a highly nutritional low caloric food that requires relatively little effort to prepare. It is full of nutrients like vitamin A, vitamin C, potassium, folate and fiber—all of which support a healthy metabolism. Zucchini, grows well in warm climates. This readily available vegetable can also be an important part of weight loss efforts. 

As to triterpenoids from *Cucurbita pepo* L, Appendino reported 3α-*p*-aminobenzoyl, 29-benzoylmultiflor-8-en-7β-ol, and 3α-*p*-aminobenzoylmultiflora-7:9(11)-dien-29-benzoate [1,2]. Barker reported large-scale isolation of bryonolic acid (3β-hydroxymultiflor-8-en-29-oic acid) [3].Wang reported cucurbitacin glycoside [4], hexanorcucurbitane glycosides [5], and purine-containing cucurbitane triterpenoids [6], extracted from *Cucurbita pepo* cv *dayangua*. Ding *et al.*, reported cerebroside, 13(18)-oleanen-3-ol, β-daucosterol, β-sitosterol, stigmasterol, dotriacontyl stearate, and tritriacontane from *Cucurbita pepo* cv *dayangua *[7]. Shibuya reported the biosynthesis of sterols and triterpenes in higher plants: *Panax ginseng*, *Olea europaea*, *Taraxacum officinale*, *Betula platyphylla*, *Glycyrrhiza glabra*, *Luffa cylindrica*, *Pisum sativum*, *Allium macrostemon*, and* Cucurbita pepo *[8]. Shibuya *et al*., also reported that three oxidosqualene cyclas (OSC) cDNAs (CPX, CPQ, CPR) were cloned from seedlings of *Cucurbita pepo* by a homology-based PCR method [9]. Careful examination of the seeds of *Cucurbita pepo* L. has led to the isolation of three novel multiflorane triterpenoids **1**–**3** along with the known compounds **4** and **5**. The structures of **1**–**3** were determined on the basis of NMR spectroscopy, including 1D and 2D (^1^H, ^1^H-COSY, NOESY, HSQC, HMBC) NMR, and EIMS.

## 2. Results and Discussion

**Figure 1:**
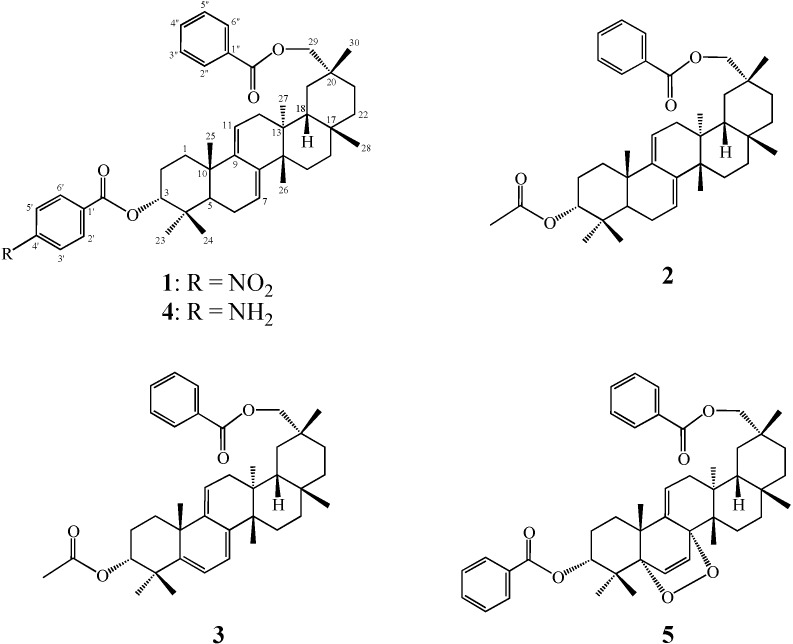
Structures for compounds **1**–**5**.

The seeds of *Cucurbita pepo *L. were extracted with MeOH and the extract was partitioned Et_2_O and H_2_O. The Et_2_O soluble portion (216.1 g) was subjected to silica gel column chromatography, medium-pressure liquid chromatography (MPLC), and normal-phase high performance liquid chromatography (HPLC) to yield five triterpenoids **1**–**5** (Figure 1) Compounds **4** and **5** were identified as 3α-*p*-aminobenzoylmultiflora-7:9(11)-dien-29-benzoate [1,2] and 5α,8α-peroxymultiflora-6:9(11)-diene-3α,29-dibenzoate [10], respectively by comparison of their characterization data with literature data.

**Figure 2:**
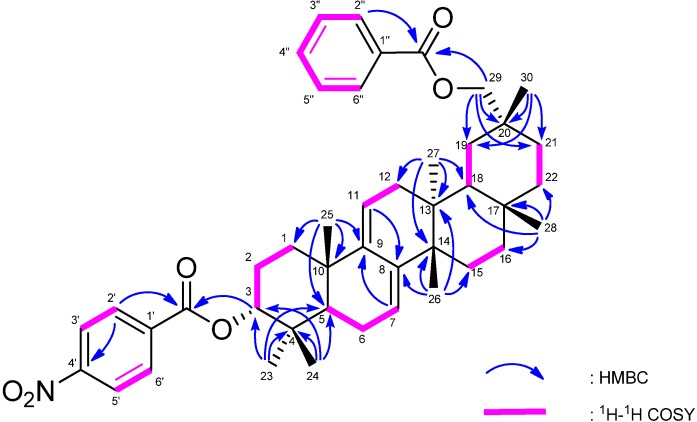
Selected ^1^H-^1^H COSY and HMBC correlations for **1**.

**Figure 3:**
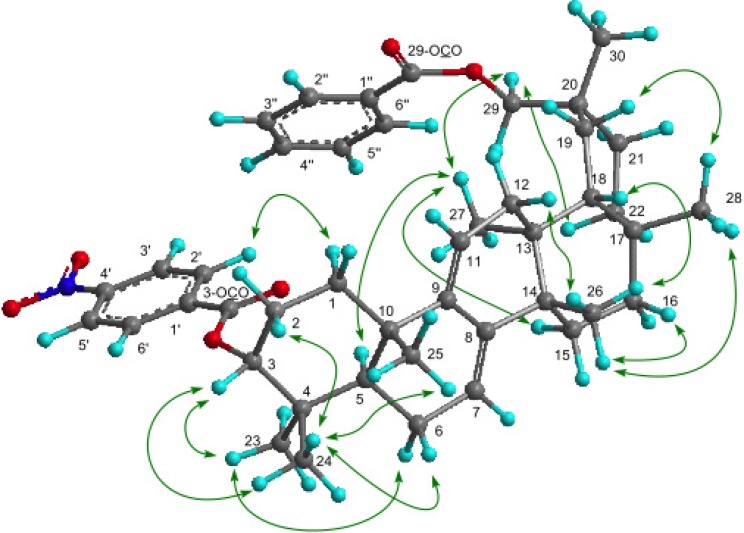
Key NOE correlations for **1**.

The molecular formula of **1** was determined as C_44_H_55_NO_6_ (M^+^; *m/z* 693.4024) based on HREIMS. The UV spectrum showed a heteroannular diene moiety (λ_max_ 230, 237, 248 nm, log ε 3.85, 3.80, 3.63). The IR spectrum showed bands assignable to ester groups (ν_max_ 1713, 1287 cm^−1^) and a nitro group (ν_max_ 1527, 1341 cm^-1^). The ^1^H- and ^13^C-NMR spectra (Table 1) exhibited signals assignable to seven tertiary methyls, ten CH_2_ groups including an oxymethylene [δ_H_ 4.11 and 4.17 (each 1H, d)], three *sp*^3^ methine groups including an oxymethine [δ_H_ 4.97 (1H, t)], two trisubstituted olefin [δ_H_ 5.24 (1H, brd); 5.53 (1H, brs)], six *sp*^3^ quaternary carbons, a benzoyl group [δ_H_ 7.43 (2H, tt), 7.52 (1H, tt), 8.08 (2H, dd); δ_C_ 128.6 (C-3″, C-5″), 129.7 (C-2″, C-6″), 130.8 (C-1″), 133.0 (C-4″), 166.9 (OCO)] and a *p*-nitrobenzoyl group [δ_H_ 8.10 (2H, dt), 8.20 (2H, dt); δ_C_ 123.8 (C-3′, C-5′), 130.7 (C-2′, C-6′), 136.3 (C-1′), 150.6 (C-4′), 164.4, (OCO)]. In the HMBC spectrum (Figure 2), long-range correlations were observed between Me-23 and C-3 (δ_C_ 80.7), C-4, C-5, and C-24; between Me-24 and C-3, C-4, C-5, and C-23; between Me-25 and C-1, C-5, C-9 (δ_C_ 144.6), and C-10; between Me-26 and C-8 (δ_C_ 142.3), C-13, C-14, and C-15; between Me-27 and C-12, C-13, C-14, and C-18; between Me-28 and C-16, C-17, C-18, and C-22; between Me-30 and C-19, C-20, C-21, and C-29 (δ_C_ 73.0); between H_2_-29 and C-19, C-20, C-21, and C-30; between H-2′ and H-6′ [δ_H_ 8.10 (2H)] and C-4′ (δ_C_ 150.6), O-C=O (δ_C_ 164.4); and between H-2″ and H-6″ [δ_H_ 8.08 (2H)] and O-C=O (δ_C_ 166.9). In the ^1^H-^1^H COSY spectrum (H-7–H_2_-6; H-11–H_2_-12; H-3–H_2_-2; H-3′ and H-5′– H-2′and H-6′; H-3″and H-5″–H-2″, H-6″ and H-4″) of** 1 **revealed the partial structures shown in bold face in Figure 2. EIMS showed a molecular ion peak at m/z 693 which is 30 mass units bigger than that of **4**. Furthermore, the same base ion peak was observed in compounds **1** and **4** at *m/z* 526 [M–*p*-nitrobenzoic acid in **1**; M–*p*-aminobenzoic acid in **4**]. On the basis of the above spectral data, **1** was established to be a novel 3α-*p*-nitrobenzoylmultiflora-7:9(11)-dien-29-benzoate. Selected NOESY correlations for **1** are shown in Figure 3. The configuration of the *p*-nitrobenzoyl group at C-3 was established as the α (axial)-orientation due to the NOE correlations between H-3 and Me-23 and Me-24, and the coupling constants of H-3 [δ_H_ 4.97 (t, *J*_3β.2α;3β,2β_ = 2.7 Hz)]. The benzoyl group was at C-29 because the NOESY correlation was observed between H_2_-29 and H-22α and Me-27. Therefore, **1** was determined as 3α-*p*-nitrobenzoylmultiflora-7:9(11)-dien-29-benzoate. Although, natural products containing nitro groups have been isolated from plants [11], e.g., monocyclic aromatic compounds [12], multicyclic aromatic compounds [13], amino acids and peptides [14], carbohydrates [15], aliphatic compounds [16], and *O*-nitro and *N*-nitro compounds [17], compound **1** is the first example which has a nitro group in triterpenoids.

**Table 1:** ^1^H- (500 MHz) and ^13^C-NMR (125 MHz) spectroscopic data of compounds **1**–**3** (CDCl_3_) ^a^.

Position	1				2				3			
	δ_H_ (*J* in Hz)		δ_C_		δ_H_ (*J* in Hz)		δ_C_		δ_H_ (*J* in Hz)		δ_C_	
1α	1.78	m	30.8	(*t*)	1.67	m	30.2	(*t*)	1.76	td (14.0, 4.3)	30.3	(*t*)
1β	1.62	m			1.48	m			1.72	m		
2α	1.92	m	23.3	(*t*)	1.73	m	23.0	(*t*)	1.82	m	22.8	(*t*)
2β	2.07	m			1.92	m			2.06	m		
3	4.97	t (2.7)	80.7	(*d*)	4.67	t (2.7)	78.2	(*d*)	4.75	dd(3.5, 2.4)(3.0)	77.4	(*d*)
4			37.4	(*s*)			36.1	(*s*)			38.9	(*s*)
5	1.77	m	43.8	(*d*)	1.63	d (4.9)	42.8	(*d*)			148.9	(*s*)
6α	2.21	m	24.0	(*t*)	2.12	brt (4.9)	23.7	(*t*)	5.85	d (6.4)	118.1	(*d*)
6β	2.21	m			2.02	m						
7	5.53	brs	118.3	(*d*)	5.49	brs	118.1	(*d*)	5.61	d (6.4)	114.4	(*d*)
8			142.3	(*s*)			141.8	(*s*)			141.2	(*s*)
9			144.6	(*s*)			144.3	(*s*)			144.6	(*s*)
10			36.4	(*s*)			36.1	(*s*)			39.2	(*s*)
11	5.24	brd (4.5)	114.3	(*d*)	5.20	d (5.3)	113.8	(*d*)	5.34	dt (5.0, 2.1)	118.4	(*d*)
12α	2.10	m	39.6	(*t*)	2.07	m	39.3	(*t*)	2.16	dd (17.4, 6.2)	40.1	(*t*)
12β	1.77	dd (11.7, 4.8)			1.75	m			1.83	m		
13			37.5	(*s*)			37.3	(*s*)			38.2	(*s*)
14			40.3	(*s*)			40.0	(*s*)			40.0	(*s*)
15α	1.85	m	27.6	(*t*)	1.71	m	27.3	(*t*)	1.77	m	26.8	(*t*)
15β	1.42	m			1.37	m			1.36	m		
16α	1.69	m	36.8	(*t*)	1.70	m	36.6	(*t*)	1.74	m	36.6	(*t*)
16β	1.52	m			1.50	t (3.8)			1.52	dt (10.0, 3.1)		
17			31.8	(*s*)			31.5	(*s*)			31.6	(*s*)
18	1.65	m	44.8	(*d*)	1.65	m	44.6	(*d*)	1.7	dd (9.2, 2.6)	44.6	(*d*)
19α	1.81	m	28.6	(*t*)	1.82	m	28.3	(*t*)	1.84	m	27.9	(*t*)
19β	1.61	m			1.54	m			1.55	m		
20			31.9	(*s*)			31.6	(*s*)			31.6	(*s*)
21α	1.48	2H, m	30.2	(*t*)	1.58	2H, m	30.0	(*t*)	1.46	m	30.1	(*t*)
21β									1.60	m		
22α	1.79	m	34.4	(*t*)	1.80	dd (10.1, 4.4)	34.0	(*t*)	1.81	m	34.0	(*t*)
22β	0.94	m			0.94	m			0.95	dt (13.8, 3.1)		
23	0.92	s	27.7	(*q*)	0.84	s	22.0	(*q*)	1.08	s	26.8	(*q*)
24	1.07	s	22.2	(*q*)	0.98	s	27.2	(*q*)	1.22	s	31.6	(*q*)
25	0.99	s	20.7	(*q*)	0.93	s	20.4	(*q*)	1.17	s	30.7	(*q*)
26	0.95	s	22.1	(*q*)	0.93	s	21.7	(*q*)	1.04	s	21.0	(*q*)
27	0.91	s	19.7	(*q*)	0.88	s	19.6	(*q*)	0.83	s	19.8	(*q*)
28	1.13	s	31.3	(*q*)	1.12	s	31.0	(*q*)	1.13	s	31.1	(*q*)
29A	4.11	d (10.7)	73.0	(*t*)	4.10	d (10.7)	72.8	(*t*)	4.08	d (10.9)	72.6	(*t*)
29B	4.17	d (10.7)			4.15	d (10.7)			4.16	d (10.9)		
30	1.12	s	30.6	(*q*)	1.11	s	30.5	(*q*)	1.11	s	30.7	(*q*)
3-OCO			164.4	(*s*)			170.9	(*s*)			171.0	(*s*)
1'			136.3	(*s*)	2.03	s	21.3	(q)	2.00	s	21.3	(*s*)
2', 6'	8.10	dt (8.9, 2.1)	130.7	(*d*)								
3', 5'	8.20	dt(8.9,2.1)	123.8	(*d*)								
4'			150.6	(*s*)								
29-OCO			166.9	(*s*)			166.7	(*s*)			166.7	(*s*)
1''			130.8	(*s*)			130.6	(*s*)			130.6	(*s*)
2'', 6''	8.08	2H, dd (7.4,2.1)	129.7	(*d*)	8.08	2H, dd (7.4,1.4)	129.4	(*d*)	8.07	2H, dd (8.2, 1.2)	129.5	(*d*)
3'', 5''	7.43	2H, tt (7.4,2.1)	128.6	(*d*)	7.45	2H, tt (7.4,1.4)	128.4	(*d*)	7.45	2H, tt (8.2,1.2)	128.4	(*d*)
4''	7.52	tt (7.4,2.1)	133.0	(*d*)	7.58	tt (7.4,1.4)	132.8	(*d*)	7.56	tt (8.2,1.2)	132.8	(*d*)

^a^Assignments were based on ^1^H-^1^H COSY, HMQC, HMBC and NOESY spectroscopic data.

**Figure 4:**
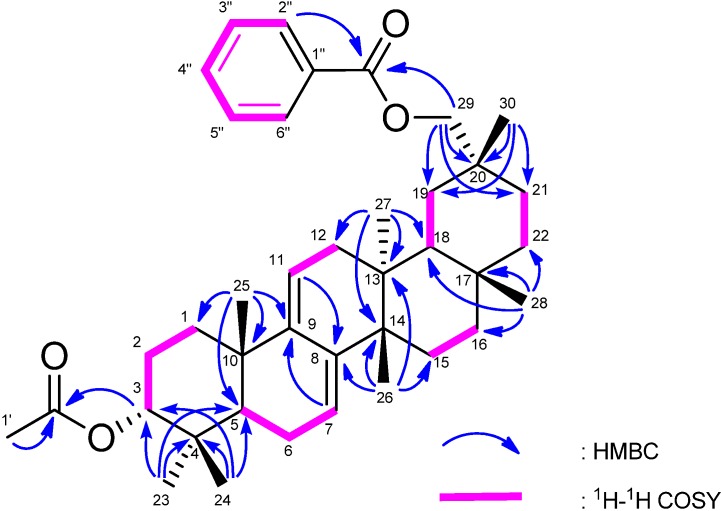
Selected ^1^H-^1^H COSY and HMBC correlations for 2.

Compound **2** was assigned the molecular formula C_39_H_54_O_4_ (M^+^; *m/z* 586.4016) based on HREIMS. The UV absorption band showed a heteroannular diene (λ_max_ 222, 237 nm, log ε 3.93, 3.95). The IR spectrum showed the presence of ester groups (ν_max_ 1743, 1718, 1271 cm^-1^). The ^1^H- and ^13^C-NMR spectra (Table 1) exhibited signals assignable to seven tertiary methyls, ten CH_2_ groups including an oxymethylene [δ_H_ 4.10, 4.15 (each 1H, d)], three *sp*^3^ methine groups including an oxymethine [δ_H_ 4.67 (1H, t)], Δ7,9(11)-diene (δ_H_ 5.20, 5.49), an acetyl group [δ_H_ 2.03 (3H, s)], and a benzoyl group [δ_H_ 7.45 (2H, tt), 7.58 (1H, tt), 8.08 (2H, dd); δ_C_ 128.4 (C-3″, C-5″), 129.4 (C-2″, C-6″), 130.6 (C-1″), 132.8 (C-4″), 166.7 (OCO)]. In the HMBC spectrum of **2** (Figure 4), long-range correlations were observed between Me-25 (δ_H_ 0.93) and C-9 (δ_C_ 144.3); between Me-26 (δ_H_ 0.93) and C-8 (δ_C_ 141.8); between Me-23 (δ_H_ 0.84) and Me-24 (δ_H_ 0.98) and C-3 (δ_C_ 78.2); and between Me-30 (δ_H_ 1.11) and C-29 [δ_C_ 72.8 (t)]. The spectral data indicated **2** to be a novel 3α-acetoxymultiflora-7:9(11)-diene-29-benzoate.

**Figure 5:**
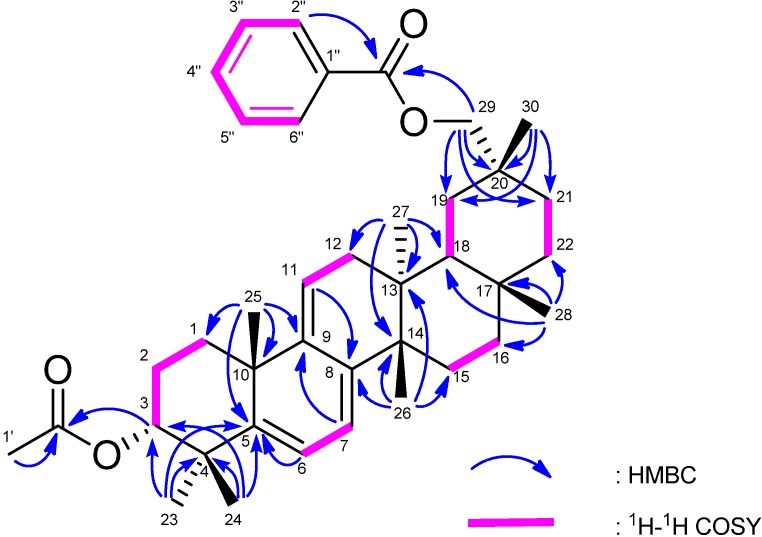
Selected ^1^H-^1^H COSY and HMBC correlations for **3**.

The molecular formula of 3 was determined as C_39_H_52_O_4_ (M^+^; *m/z* 584.3864) based on HREIMS. The UV spectrum showed a 5(6),7,9(11)-triene moiety (λ_max_ 227, 304, 315, 334 nm, log ε 4.19, 3.98, 4.00, 3.72). The IR spectrum showed bands assignable to ester groups (ν_max_ 1725, 1239 cm^-1^). The ^1^H- and ^13^C-NMR spectra (Table 1) exhibited signals due to seven tertiary methyls, nine CH_2_ groups including an oxymethylene [δ_H_ 4.08, 4.16 (each 1H, d)], three *sp*^3^ methine groups including an oxymethine [δ_H_ 4.75 (1H, t)], three trisubstituted olefin [δ_H_ 5.34 (1H, dt); 5.61 (1H, d); 5.85 (1H, d)], six *sp*^3^ quaternary carbons, an acetyl group [δ_H_ 2.00; δ_C_ 171.0 (s)], and a benzoyl group [δ_H_ 7.45 (2H, tt), 7.56 (1H, tt), 8.07 (2H, dd); δ_C_ 128.4 (C-3″, C-5″), 129.5 (C-2″, C-6″), 130.6 (C-1″), 132.8 (C-4″), 166.7 (OCO)]. In the HMBC spectrum (Figure 5), long-range correlations were observed between Me-23 (δ 1.08) and C-3 (δ_C_ 77.4), C-4, C-5 [δ_C_ 148.9 (s)], and C-24; between Me-24 (δ_H_ 1.22) and C-3, C-4, C-5, and C-23; and between Me-25 (δ 1.17) and C-1, C-5, C-9 [δ_C_ 144.6 (s)], and C-10. In the ^1^H-^1^H COSY spectrum, H-6 (δ_H_ 5.85) correlated with only H-7 (δ_H_ 5.61); H-11 (δ_H_ 5.34) correlated with H_2_-12 (δ_H_ 1.83, 2.16). EIMS showed a fragment ion peak at *m/z* 524 [M–AcOH]^+^ as a base ion peak. Based on the spectral data, the structure of **3** was established as 3α-acetoxymultiflora-5(6):7:9(11)-trien-29-benzoate.

Compounds **1**–**5** were evaluated for cytotoxic activity against HL-60 and P388 cells using MTT methods (Table 2) [18]. Although, **2** exhibited weak cytotoxic activity against HL-60 (IC_50_ 25.7 μM) and P388 (IC_50_ 75.1 μM), **1** and **3**–**5** showed no activity against either cell line. Compound **3** showed melanogenesis inhibitory activity with low cytotoxicity at 100 μM (melanin content 66.9%, cell viability 92.5%) (Table 3). Compound **2** exhibited strong melanogenesis inhibitory activity, although probably due to its cytotoxic action (cell viability 32.8%, 69.3%, and 87.6% at 100, 30, and 10 μM, respectively).

**Table 2:** Cytotoxic activity of multiflorane-type triterpenes from *Cucurbita pepo* seeds.

Compound	IC_50_ (μM)^a^	
	HL-60	P388
	(human leukemia)	(murine leukemia)
**1**	>100	>100
**2**	25.7 ± 1.1	75.1 ± 0.8
**3**	>100	>100
**4**	>100	>100
**5**	>100	>100
5-fluorouracil ^b^	2.3 ± 0.2	1.9 ± 0.2

^a^ HL-60 and P388 cell lines (each 1 × 10^4^ cells in 100 μL) were treated with test compounds for 72 h, and MTT solution was added to the wells. The grown cells were labeled with 5 mg/ml MTT in phosphate-buffered saline (PBS), and the absorbance of formazan dissolved with 20% sodium dodecyl sulfate (SDS) in 0.1 N HCl was measured at 550 nm using a microplate reader. Data are expressed as mean ± standard deviation (S.D.) (n = 3); ^b^ Reference compound.

**Table 3:** Melanogenesis inhibitory activities and cytotoxicities in B16 mouse melanoma cell line of multiflorane-type triterpenes isolated from *Cucurbita pepo* ^a^.

Compound	Mean ± S.D. (%) at 10 μM						Mean ± S.D. (%) at 30 μM						Mean ± S.D. (%) at 100 μM					
	Melanin content			Cell viability			Melanin content			Cell viability			Melanin content			Cell viability		
**1**	103.7	±	5.2	91.1	±	4.4	99.4	±	3.7	82.3	±	4.3	92.7	±	3.1	76.4	±	0.8
**2**	73.6	±	1.0	87.6	±	0.2	69.9	±	4.4	69.3	±	1.3	31.4	±	2.8	32.8	±	2.8
**3**	97.3	±	0.9	99.4	±	4.0	93.5	±	2.5	99.4	±	3.8	66.9	±	5.0	92.5	±	4.3
**4**	97.4	±	2.1	102.4	±	4.3	96.8	±	1.0	96.2	±	1.3	98.5	±	8.4	88.0	±	5.9
**5**	102.0	±	9.2	100.9	±	1.8	101.1	±	6.9	99.2	±	9.6	92.4	±	4.7	97.6	±	6.6
arbutin^b^	88.9	±	2.3	100.0	±	2.7	72.3	±	3.1	94.4	±	1.2	55.3	±	1.0	89.9	±	0.3

^a^ Melanin content (%) and cell viability (%) were determined based on the absorbances at 450 nm, and 550 nm, respectively, by comparison with those for DMSO (100%). Each value represents the mean ± S.D. of three determinations. Concentration of DMSO in the sample solution was 2 μL/mL; ^b^ Reference compound.

## 3. Experimental

### 3.1. General Procedures

Melting points were determined on a Yanagimoto micro-melting point apparatus and are uncorrected. Optical rotations were measured using a JASCO DIP-1000 digital polarimeter. IR spectra were recorded using a Perkin-Elmer 1720X FTIR spectrophotometer. ^1^H- and ^13^C-NMR spectra were obtained on a Varian INOVA 500 spectrometer with standard pulse sequences, operating at 500 and 125 MHz, respectively. CDCl_3_ was used as the solvent and TMS, as the internal standard. EIMS were recorded on a Hitachi 4000H double-focusing mass spectrometer (70 eV). Column chromatography was carried out over silica gel (70–230 mesh, Merck, Darmstadt, Germany) and MPLC was carried out with silica gel (230–400 mesh, Merck, Darmstadt, Germany). HPLC was run on a JASCO PU-1586 instrument equipped with a differential refractometer (RI 1531). Fractions obtained from column chromatography were monitored by TLC (silica gel 60 F_254_, Merck). 

### 3.2. Plant Material

The seeds of *Cucurbita pepo* L. produced in USA (California), were purchased from JA (Japan Agricultural Co-opwration)-Takatsuki in May, 2011.

### 3.3. Isolation Procedure

Air-dried seeds (10 kg) were ground and extracted × 3 for 3 days each with MeOH (10 L) employing an automatic percolator. Removal of the MeOH under reduced pressure left a greenish residue which was partitioned between Et_2_O and H_2_O. Evaporation of the Et_2_O phase gave a yellowish residue (216.1 g) which was subjected to silica gel (3.5 kg) column chromatography. Elution of the column with CHCl_3_ gave residue A (Fr. No. 1–18, 39.5 g), B (Fr. No. 19–25, 14.9 g) and C (Fr. No. 26–30, 10.6 g). Elution of the column with CHCl_3_/EtOAc (10:1) afforded residues D (Fr. No. 31–33, 21.5 g) and E (Fr. No. 34–57, 13.4 g) and subsequent column chromatography with CHCl_3_/EtOAc (2:1) to give residues F (Fr. (Fr. No. 58–68, 2.0 g). Elution was continued with EtOAc and MeOH to give residues G (Fr. No. 69–74, 2.0 g) and H (Fr. No. 75–77, 4.5 g). 

Residue B was rechromatographed on a silica gel (230–400 mesh, 500 g) column using *n*-hexane:EtOAc = 20:1~EtOAc to give residues B-1 (Fr. Nos. 28–29, 10.9 mg), B-2 (Fr. Nos. 30–33, 30.2 mg), B-3 (Fr. Nos. 34–39, 32.2 mg). Residue B-1 was separated by HPLC (Normal phase silica gel, *n*-hexane:EtOAc = 10:1) to give compounds **1** (2.5 mg), **2** (5.1 mg) and **3** (1.8 mg). 

Residue C was rechromatographed on a silica gel (230–400 mesh, 200 g) column using *n*-hexane:EtOAc = 10:1~EtOAc to give residues C-1 (Fr. Nos. 1–20, 176.4 mg), C-2 (Fr. Nos. 21–39, 60.2 mg), C-3 (Fr. Nos. 40–47, 3.4 g). Residue C-1–C-3 was separated by HPLC (Normal phase silica gel, *n*-hexane:EtOAc = 10:1) to give compounds **4** (28.3 mg) and **5** (4.9 mg). Compound **4** was identified as 3α-*p*-aminobenzoylmultiflora-7:9(11)-dien-29-benzoate on the basis of published data [1,2], and **5** was identified as 5α,9α-peroxymultiflora-6,9(11)-diene-3α,29-dibenzoate on the basis of published data [10].

### 3.4. Compound 1

Colorless crystals; mp 172–174 °C (from MeOH-CHCl_3_); [α]_D_^26^ +10.9° (*c* 0.048, CHCl_3_); HREIMS *m/z*: 693.4024 [M]^+^ (C_44_H_55_NO_6_, calcd for 693.4029); UV (EtOH) λ_max_ nm (log ε): 230 (3.85), 237 (3.80), 248 (3.63); IR (KBr) ν_max_ cm^−1^; 2945, 1713 (O-C=O), 1542 (Ar), 1527 and 1341 (NO_2_), 1510, 1371, 1287; ^1^H- and ^13^C-NMR, see Table 1. EIMS* m/z *(rel. int.): 693 (100) [M]^+^), 526 (41) [M–*p*-nitrobenzoic acid]^+^, 389 (26), 253 (71), 227 (37), 211 (37).

### 3.5. Compound 2

Colorless crystals; mp 93–94 °C (from MeOH-CHCl_3_); [α]_D_^26^–44.0° (*c* 0.11, CHCl_3_); HREIMS *m/z*: 586.4016 [M]^+^ (C_39_H_54_O_4_, calcd for 586.4022); UV (EtOH) λ_max_ nm (log ε): 222 (3.93), 237 (3.95); IR (KBr) ν_max_ cm^−1^; 2974, 1743, 1718 and 1271 (O-C=O), 1559 (Ar), 1521, 1489, 1458, 1271, 1114; ^1^H- and ^13^C-NMR, see Table 1. EIMS* m/z *(rel. int.): 586 (62) [M]^+^, 526 (100) [M–HOAc]^+^, 511 (31), 389 (35), 253 (62).

### 3.6. Compound 3

Colorless crystals; mp 105–107 °C; [α]_D_^26^–291.6° (*c* 0.255, CHCl_3_); HREIMS *m/z*: 584.3864 [M]^+^ (C_39_H_52_O_4_, calcd for 584.3866); UV λ_max_ (EtOH) nm (log ε): 227 (4.19), 304 (3.98), 315 (4.00), 334 (3.72); IR (KBr) ν_max_ cm^−1^: 2949, 2881, 1725 and 1239 (O-C=O), 1540 (Ar), 1450, 1274, 1105, 992, 773; ^1^H- and ^13^C-NMR, see Table 1. EIMS* m/z *(rel. int.): 584 (33) [M]^+^, 524 (100) [M–HOAc]^+^, 509 (52), 457 (11), 387 (35), 295 (23), 285 (36), 251 (30), 225 (51).

### 3.7. Cytotoxicity Assay

The cytotoxicity assay was determined previously [18]. Briefly, the HL-60 and P388 cell lines (each 1 × 10^4^ cells in 100 μL) were treated with test compounds for 72 h, and MTT solution was added to the wells. The grown cells were labeled with 5 mg/mL MTT in phosphate-buffered saline (PBS), and the absorbance of formazan dissolved with 20% sodium dodecyl sulfate (SDS) in 0.1 N HCl was measured at 550 nm using a microplate reader (Model 450, BioRad, Richmond, CA).

### 3.8. Determination of Cell Proliferation

Cell proliferation was examined according to a method reported previously [19]. Briefly, B16 4A5 cells [obtained from Riken Cell Bank (Tsukuba, Ibaraki, Japan)] (3 × 10^4^ cells in 500 μL), preincubated for 24 h were treated for 48 h with test samples dissolved in dimethyl sulfoxide (DMSO) at a final concentration of 100, 30 or 10 μM, and MTT solution was added. After 3 h of incubation, 2-propanol containing 0.08 M HCl was added to dissolve the formazan produced in the cells. The absorbance of each well was read at 550 nm using a microplate reader.

### 3.9. Assay of Melanin Content

The assay of melanin content was performed as described previously [19]. B16 cells were pre-incubated as above in α-MSH (100 nM) containing medium. Test samples dissolved in DMSO were added to the medium and the cells were cultured for 48 h. The medium was removed and the cells were dissolved in 2 M NaOH containing 10% DMSO. The amount of melanin was determined spectrophotometrically by measuring absorbance at 450 nm using a microplate reader. The optical density of control cells was assumed to be 100%.

## 4. Conclusions

The structure of **1** was established as 3α-*p*-nitrobenzoylmultiflora-7:9(11)-dien-29-benzoate. This is the first report of a triterpenoid having a nitro group in the molecule. At this time we have no explanation for the presence of a *p*-nitrobenzoic moiety in a zucchini metabolite, and wish to consider the role of the nitro group in the plant body in the future.

## Supplementary Materials

Supplementary materials can be accessed at: http://www.mdpi.com/1420-3049/18/7/7448/s1.
