# Supplementary material for: A Novel 3α-p-Nitrobenzoylmultiflora-7:9(11)-diene-29-benzoate and Two New Triterpenoids from the Seeds of Zucchini (Cucurbita pepo L)
Source: Molecules. 2013 Jun 26;18(7):7448–59. doi: 10.3390/molecules18077448 (PMC6269766; doi:10.3390/molecules18077448)
Supplement: Supplementary file 1 [file molecules-18-07448-s001.pdf]

Supplementary Materials

S1. ^{1}H NMR spectrum of **1**.
S2. ^{13}C NMR spectrum of **1**.
S3. HSQC spectrum of **1**.
S4. HMBC spectrum of **1**.
S5. ^{1}H-^{1}H COSY spectrum of **1**.
S6. NOESY spectrum of **1**.
S7. EIMS of **1**.
S8. HREIMS of **1**.
S9. ^{1}H NMR spectrum of **2**.
S10. ^{13}C NMR spectrum of **2**.
S11. HSQC spectrum of **2**.
S12. HMBC spectrum of **2**.
S13. ^{1}H-^{1}H COSY spectrum of **2**.
S14. NOESY spectrum of **2**.
S15. EIMS of **2**.
S16. HREIMS of **2**.
S17. ^{1}H NMR spectrum of **3**.
S18. ^{13}C NMR spectrum of **3**.
S19. HSQC spectrum of **3**.
S20. HMBC spectrum of **3**.
S21. ^{1}H-^{1}H COSY spectrum of **3**.
S22. NOESY spectrum of **3**.
S23. EIMS of **3**.
S24. HREIMS of **3**.

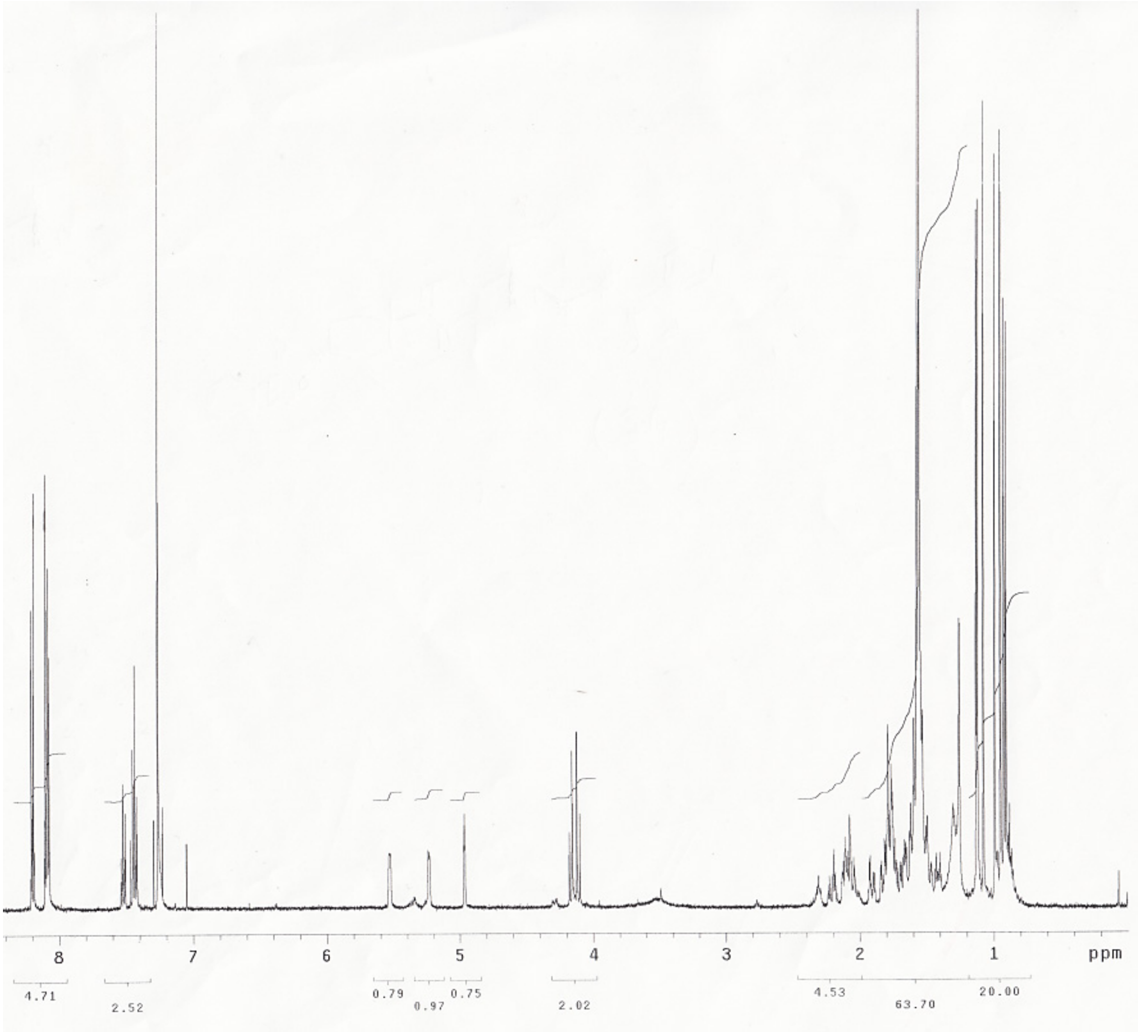

S1. ^{1}H NMR spectrum of **1**.

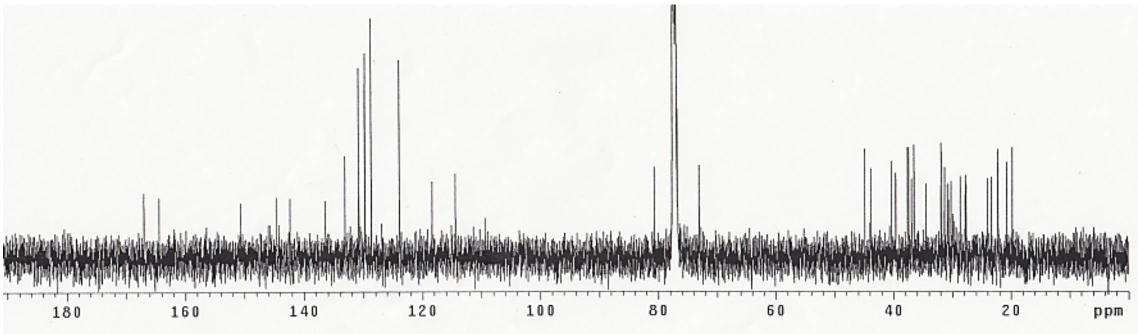

S2. ^{13}C NMR spectrum of **1**.

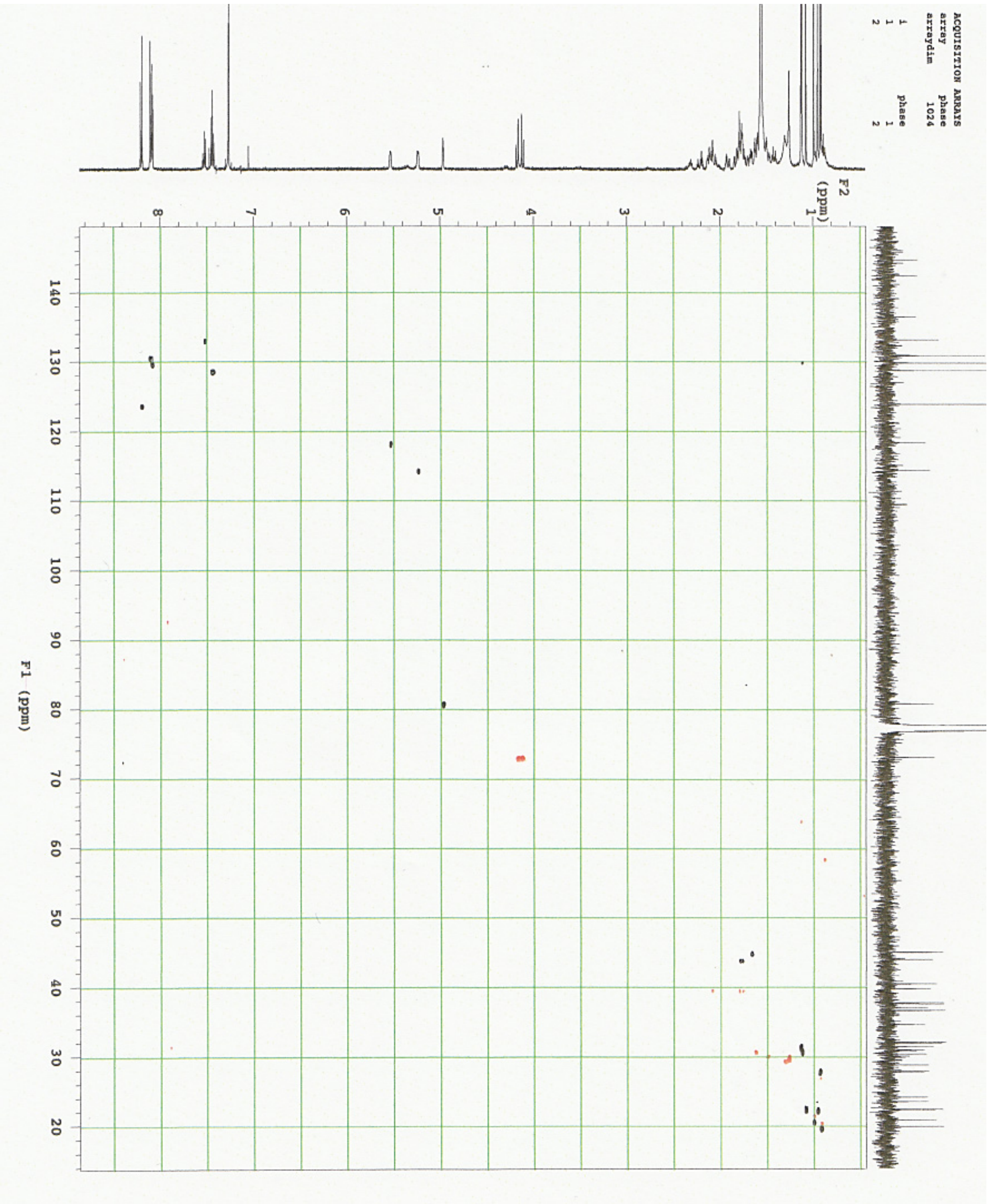

S3. HSQC spectrum of **1**.

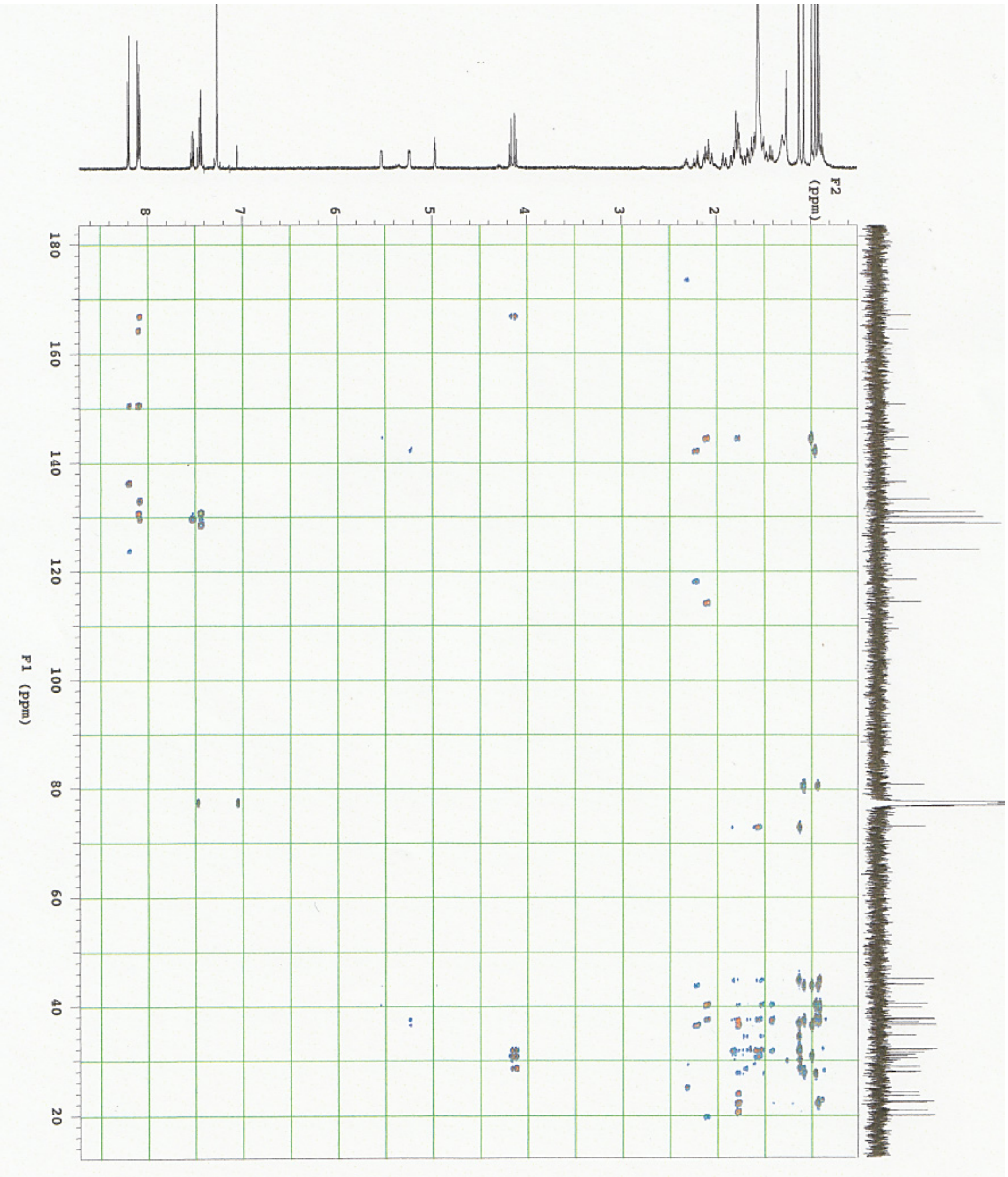

S4. HMBC spectrum of **1**.

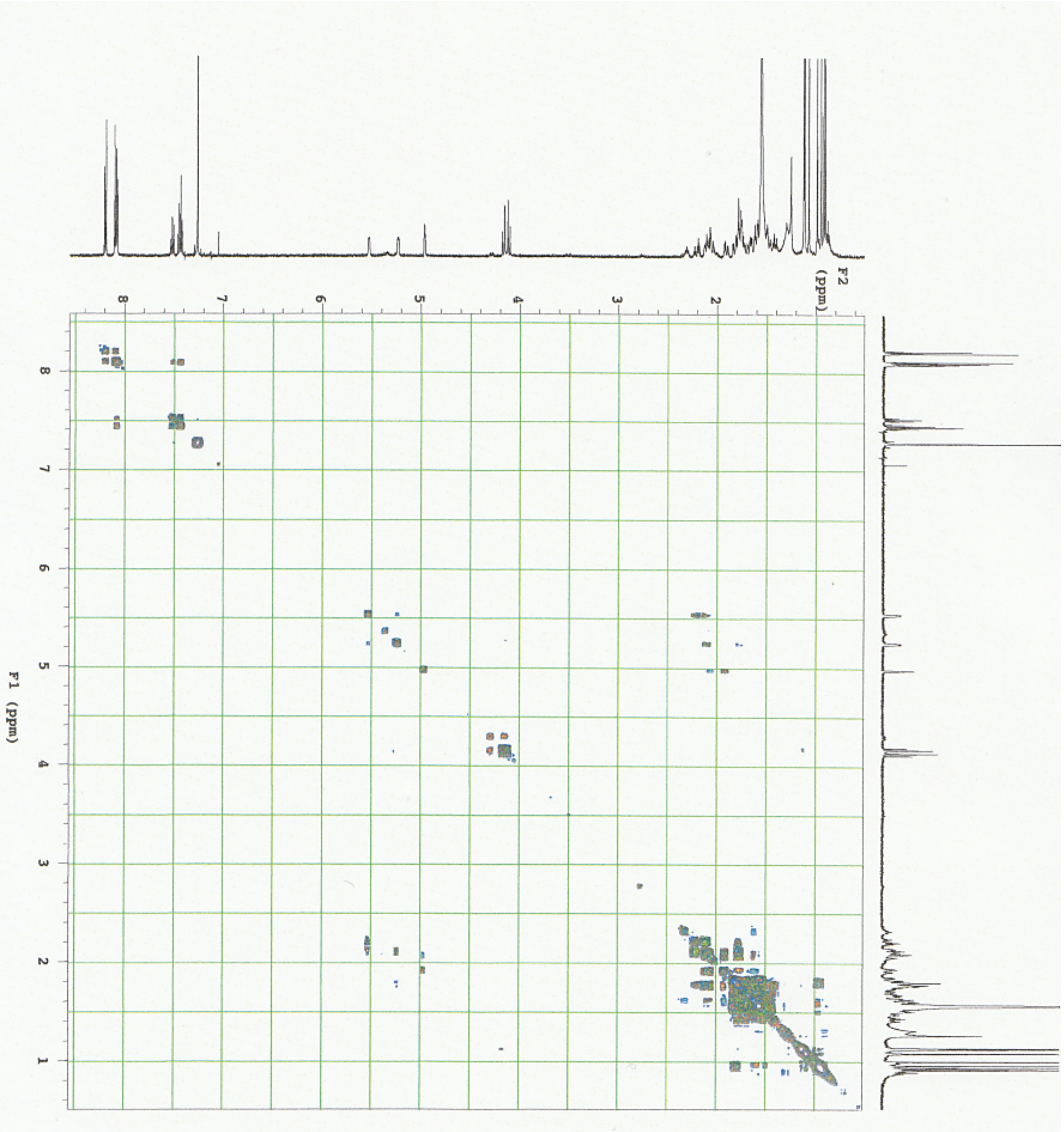

S5. ^{1}H-^{1}H COSY spectrum of **1**.

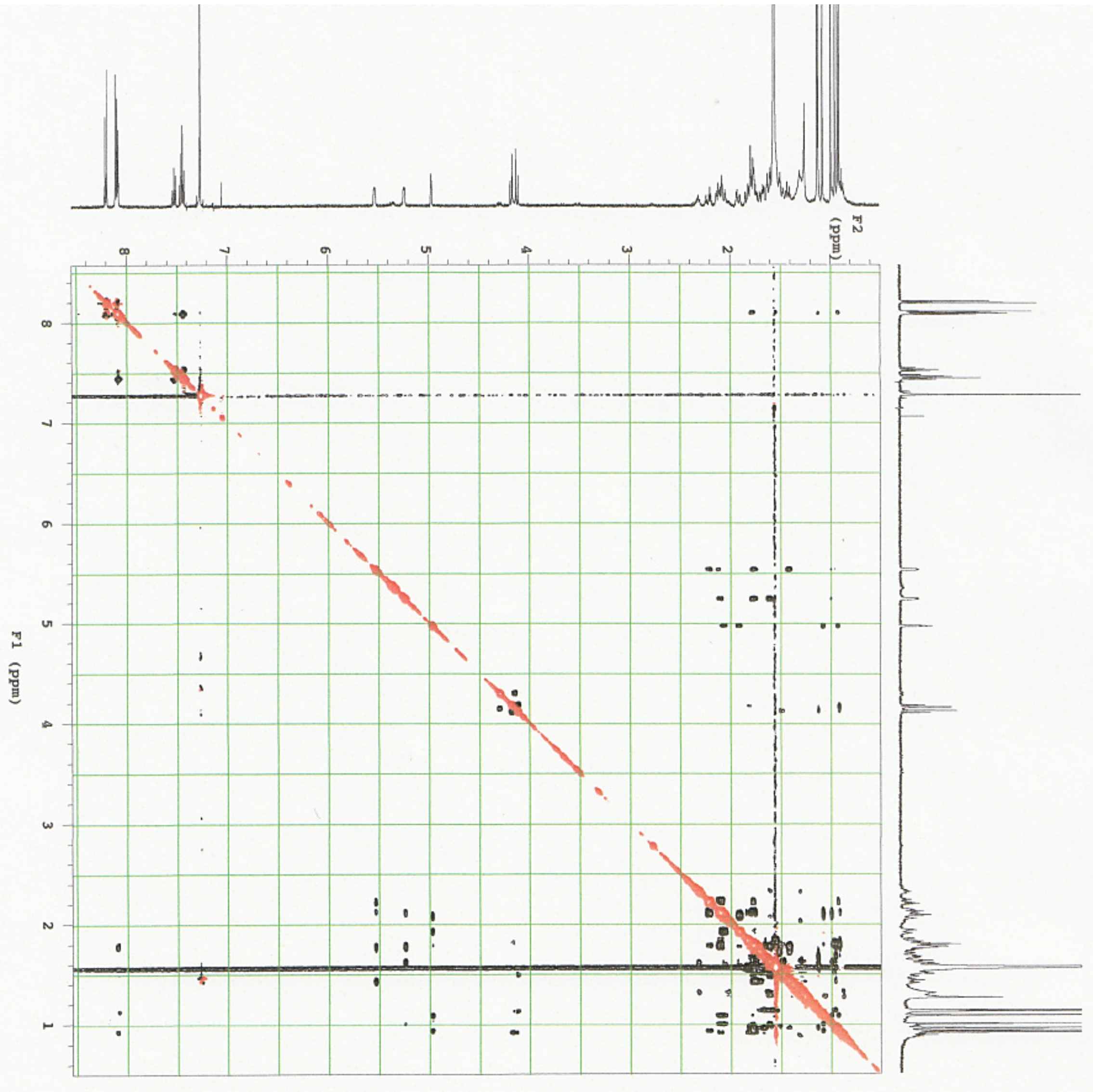

S6. NOESY spectrum of **1**.

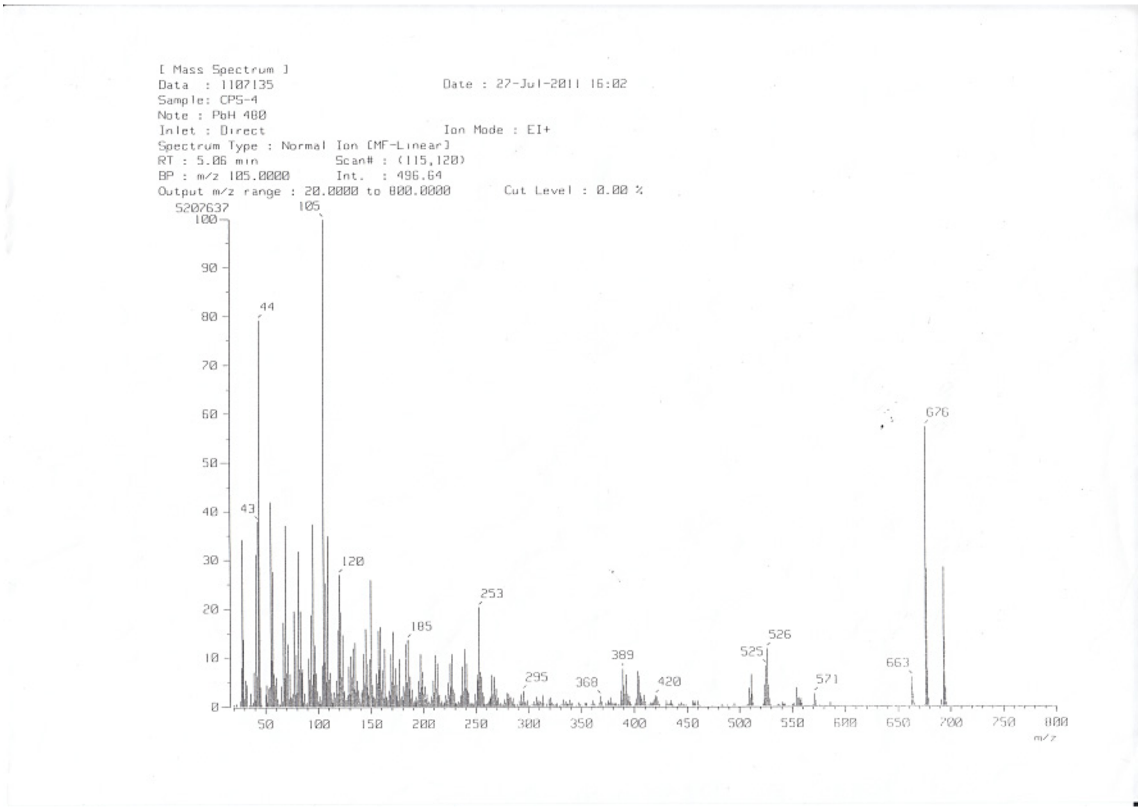

S7. EIMS of **1**.

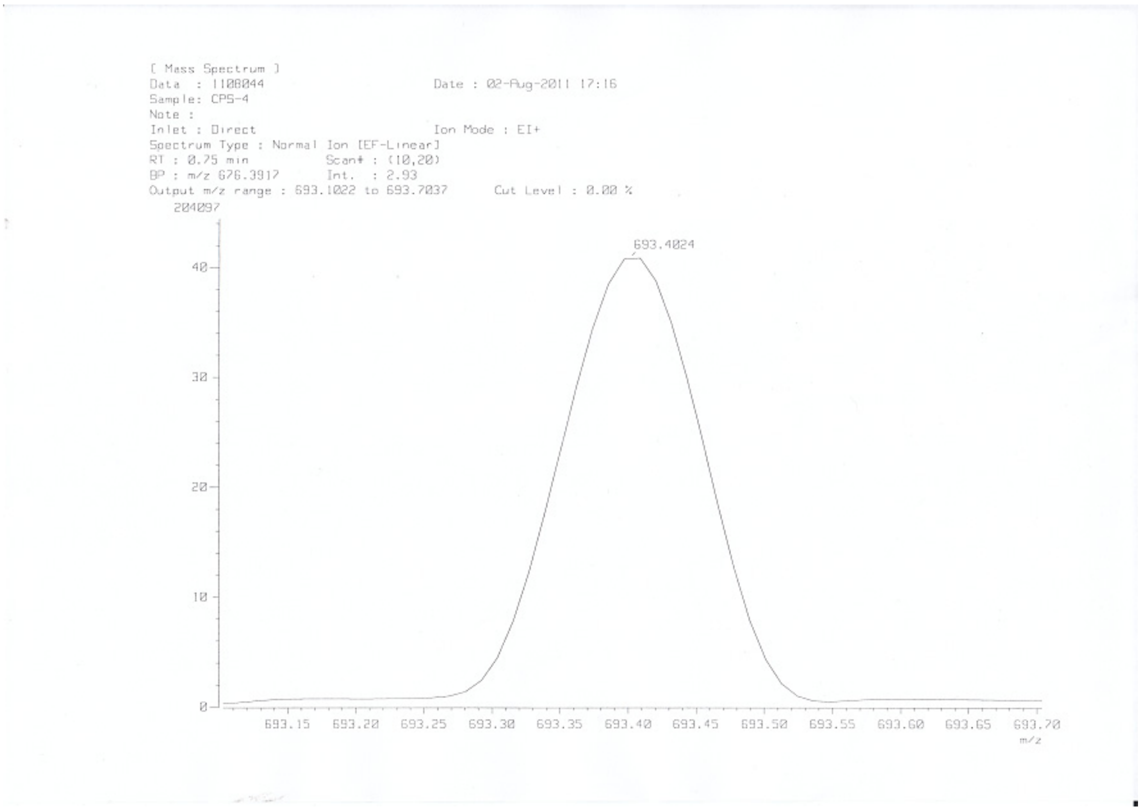

S8. HREIMS of **1**.

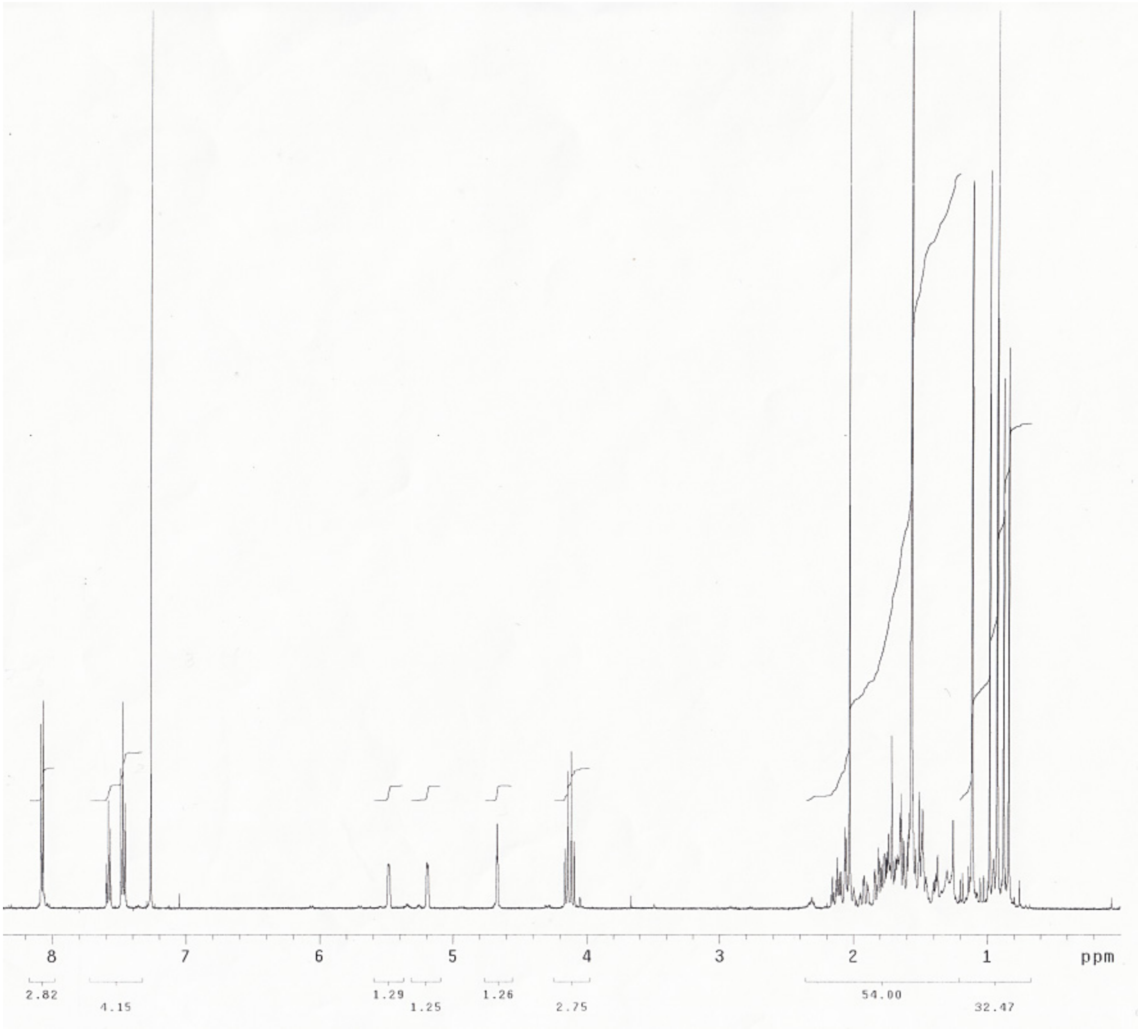

S9. ^{1}H NMR spectrum of **2**

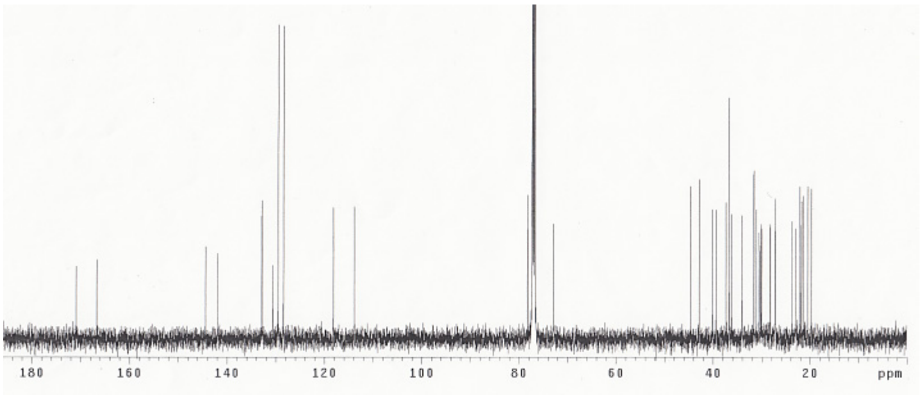

S10. ^{13}C NMR spectrum of **2**.

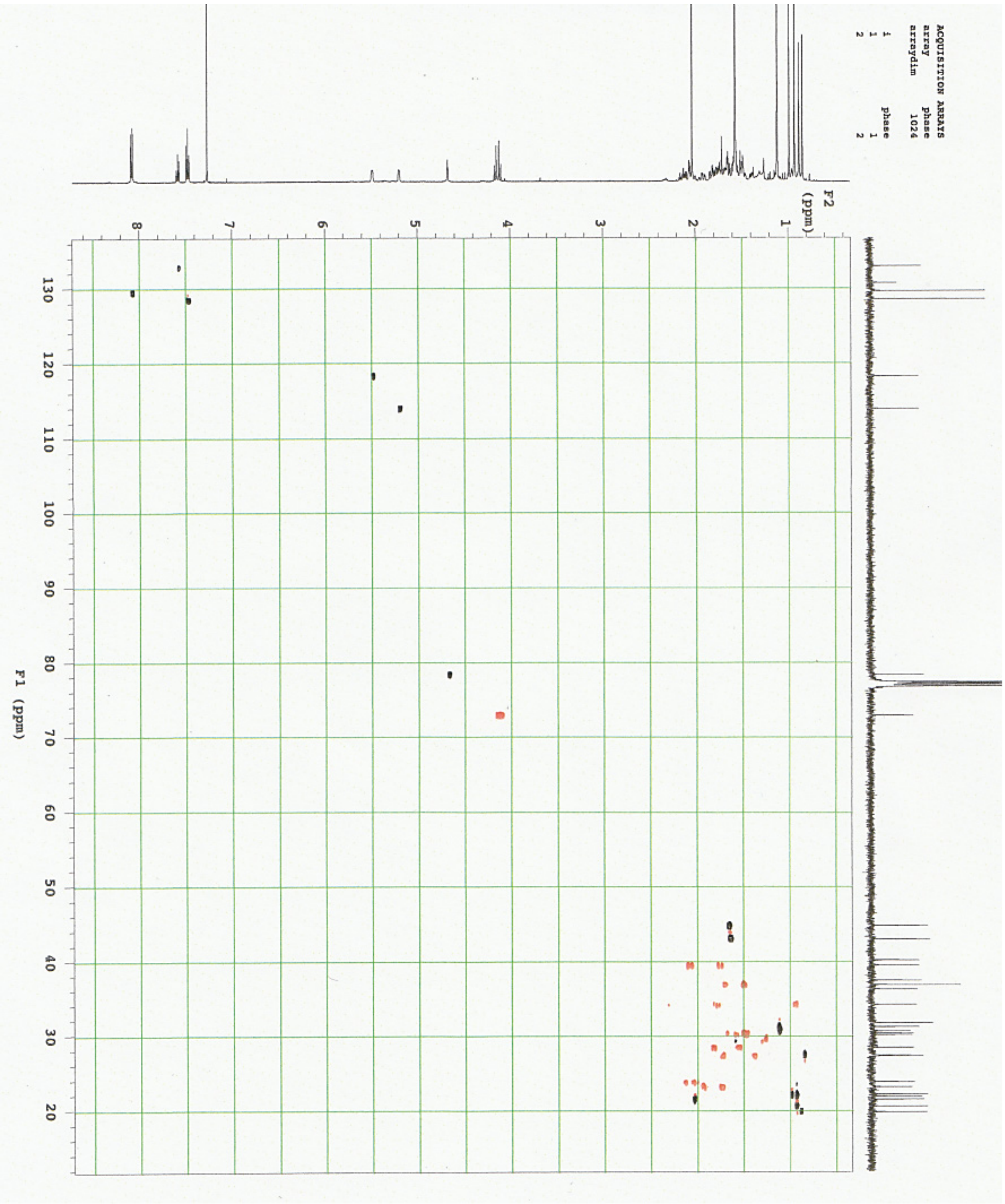

S11. HSQC spectrum of **2**.

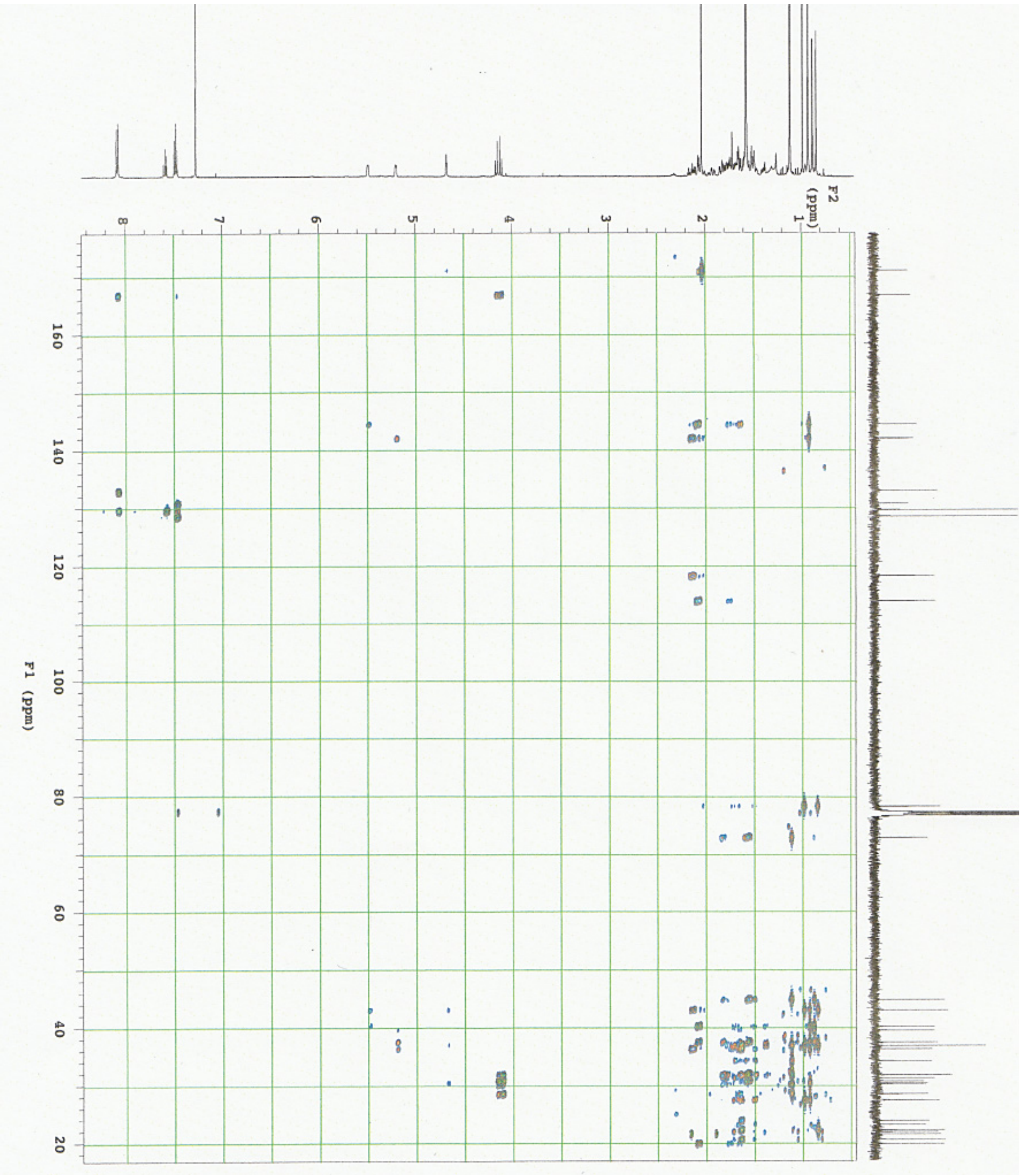

S12. HMBC spectrum of **2**.

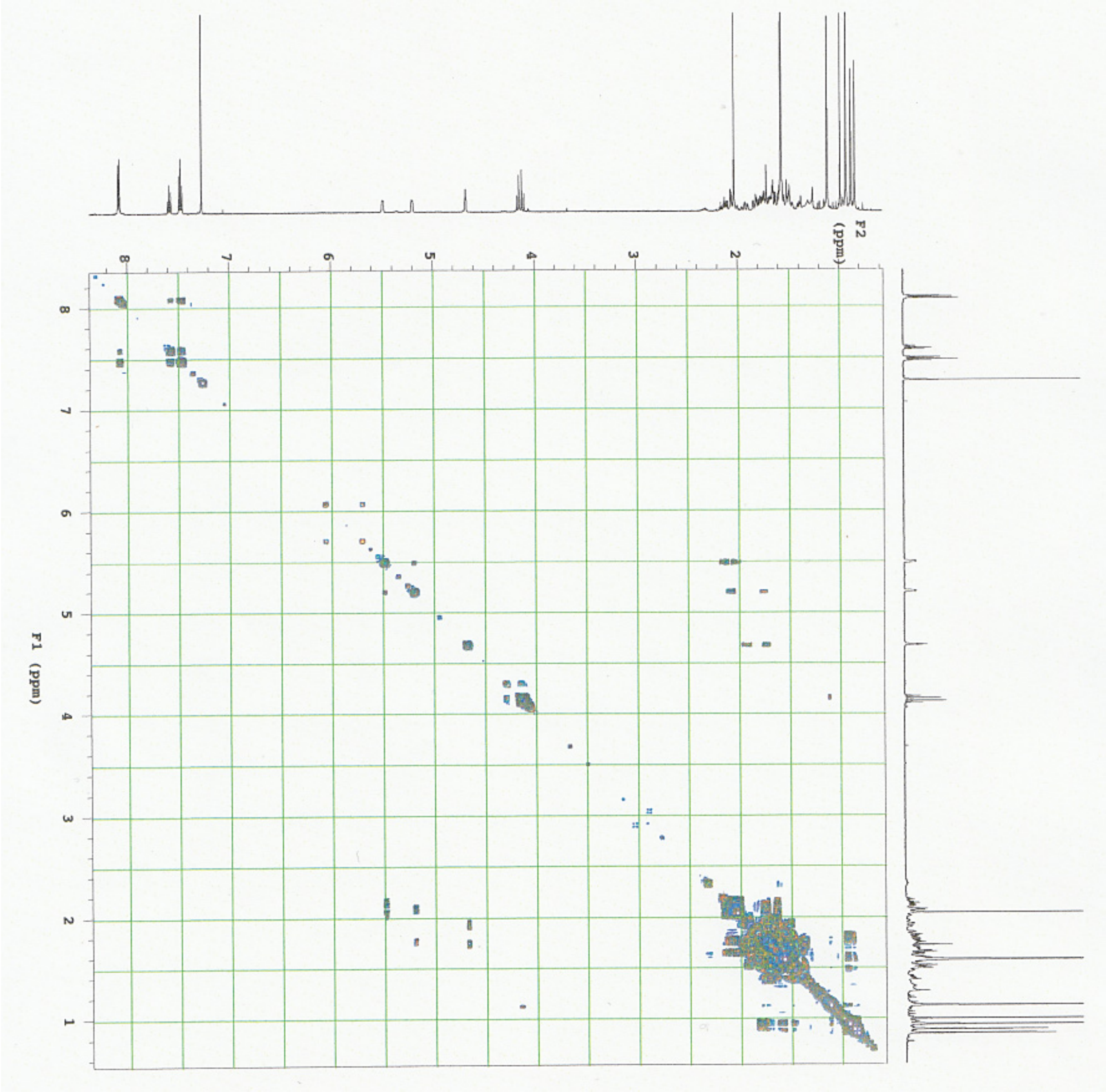

S13. ^{1}H-^{1}H COSY spectrum of **2**.

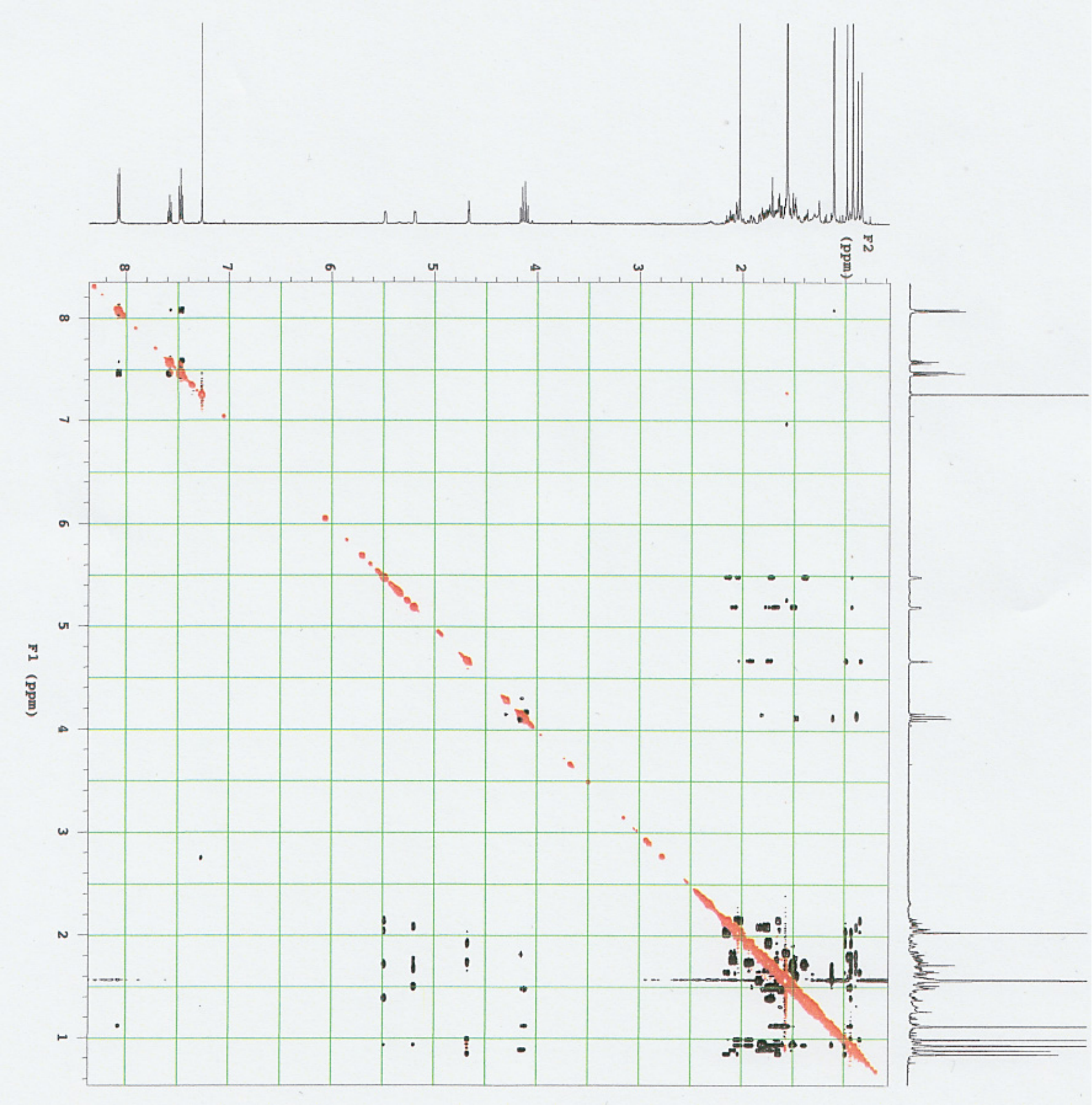

S14. NOESY spectrum of **2**.

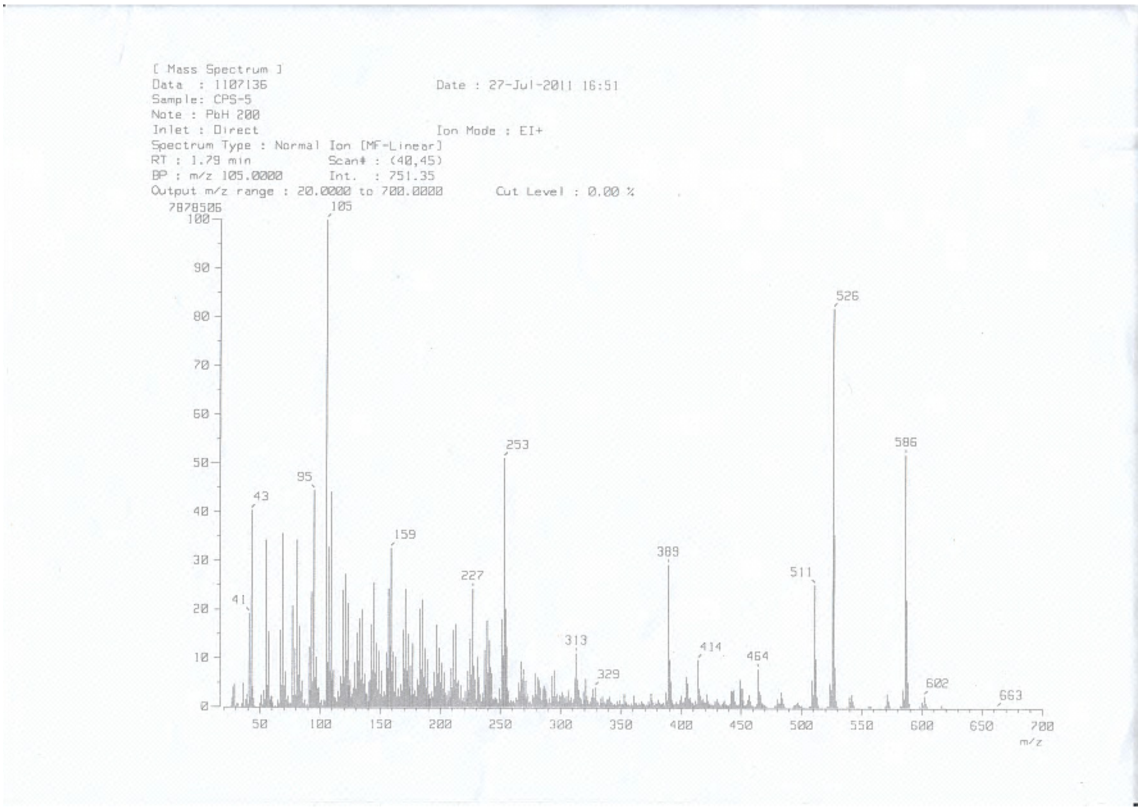

S15. EIMS of **2**.

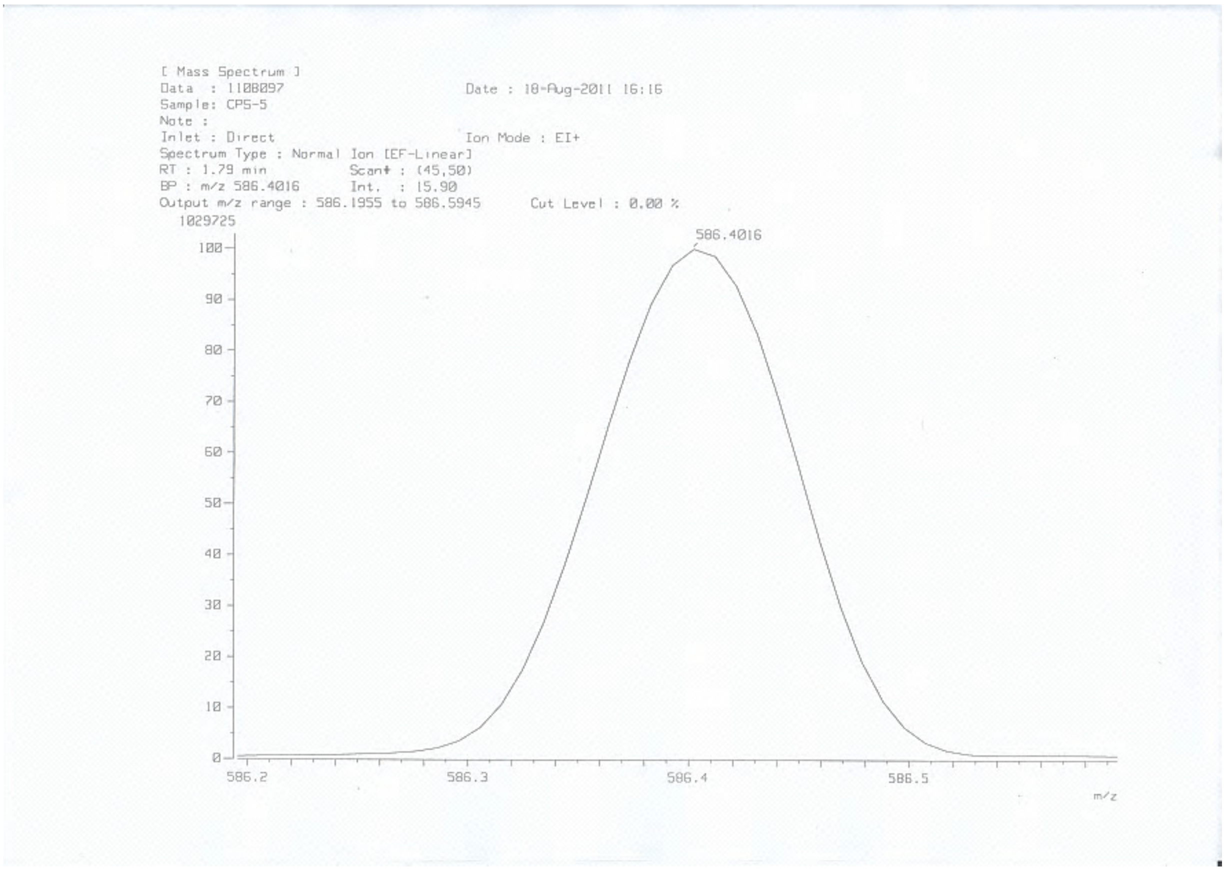

S16. HREIMS of **2**.

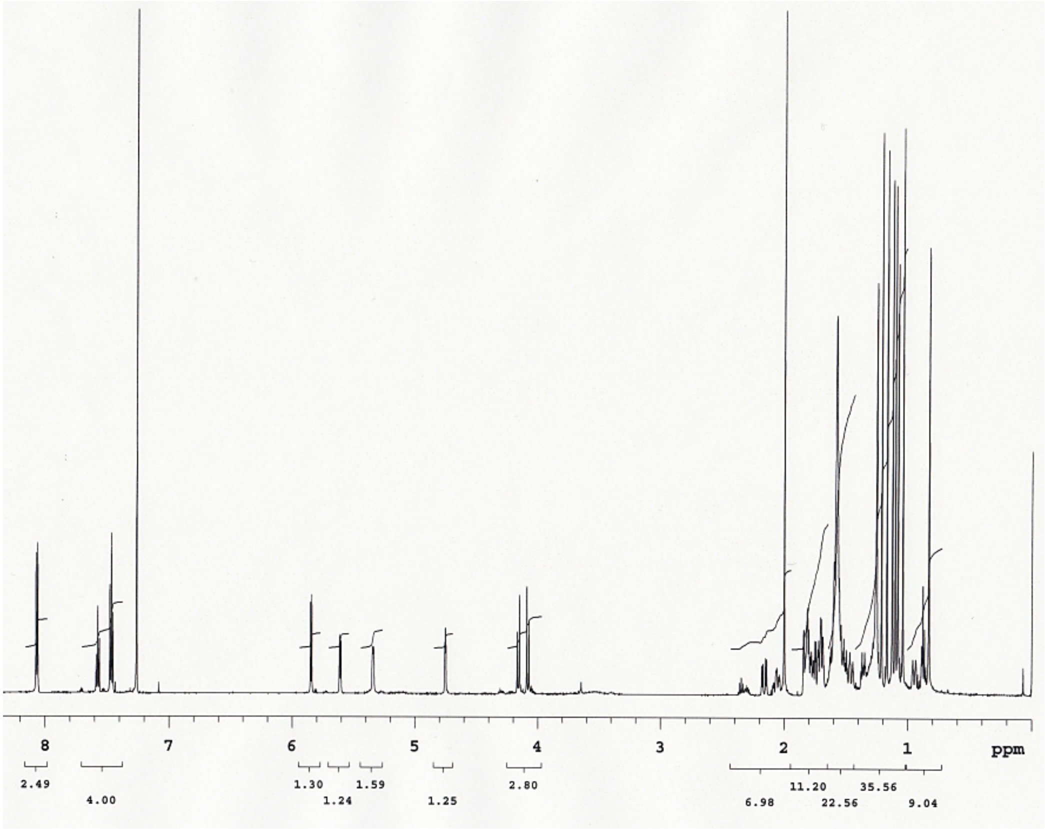

S17. ^{1}H NMR spectrum of **3**.

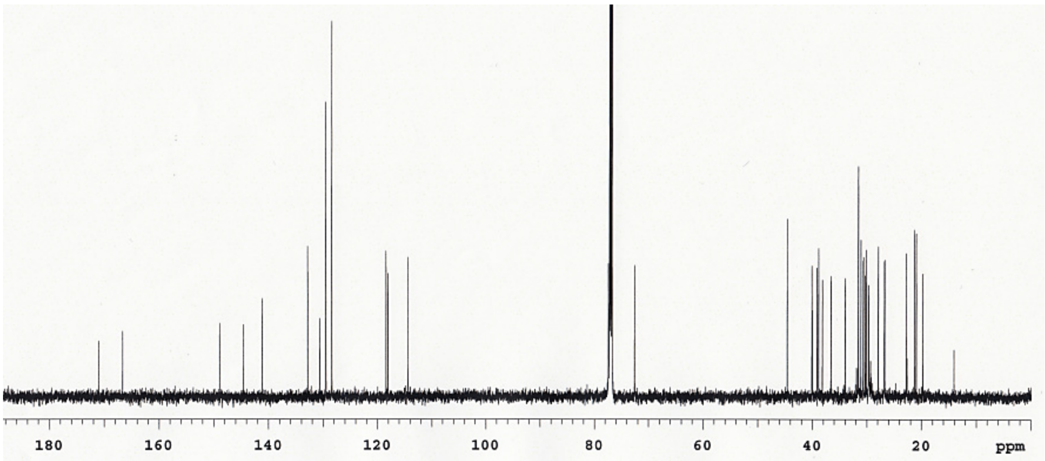

S18. ^{13}C NMR spectrum of **3**.

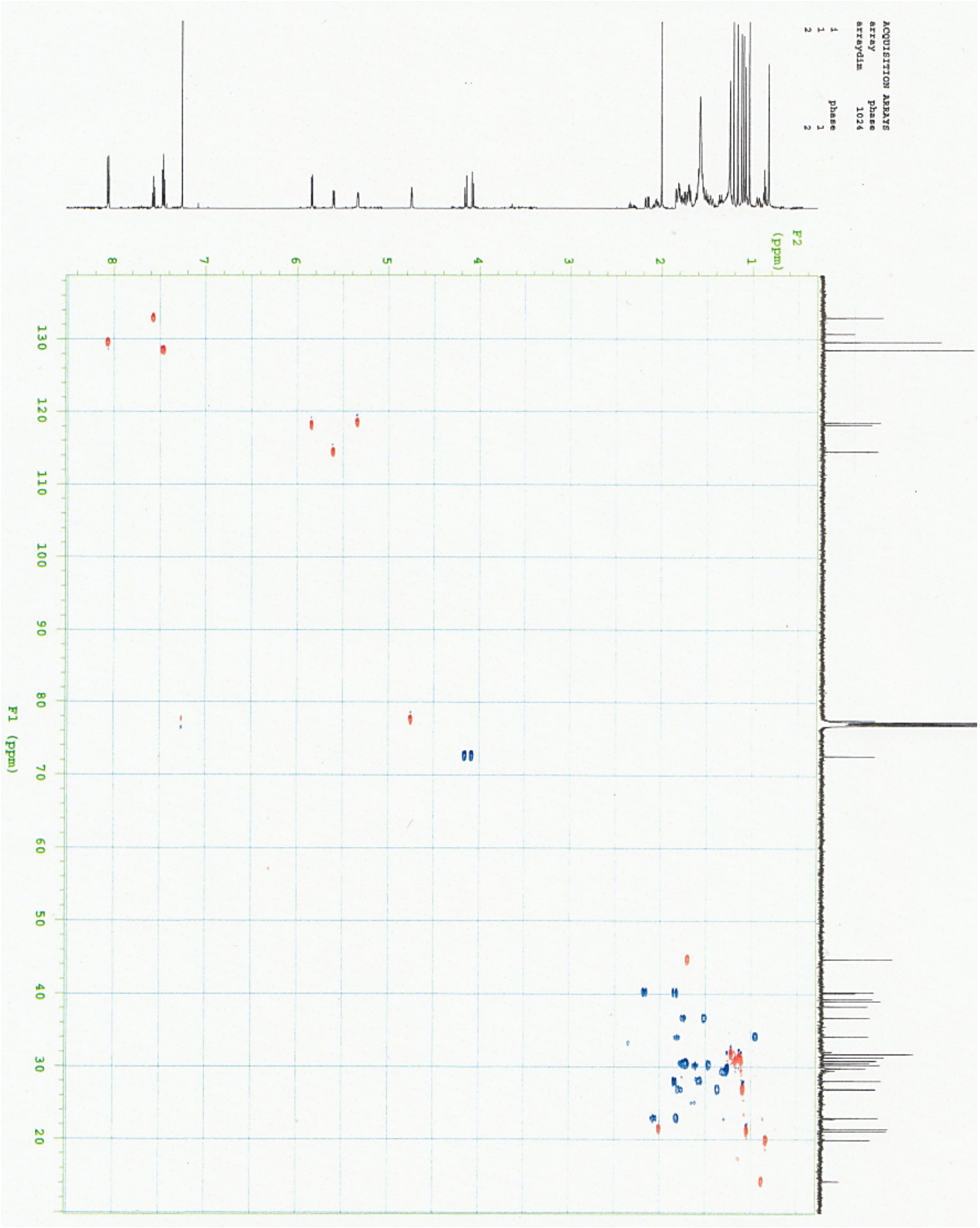

S19. HSQC spectrum of **3**.

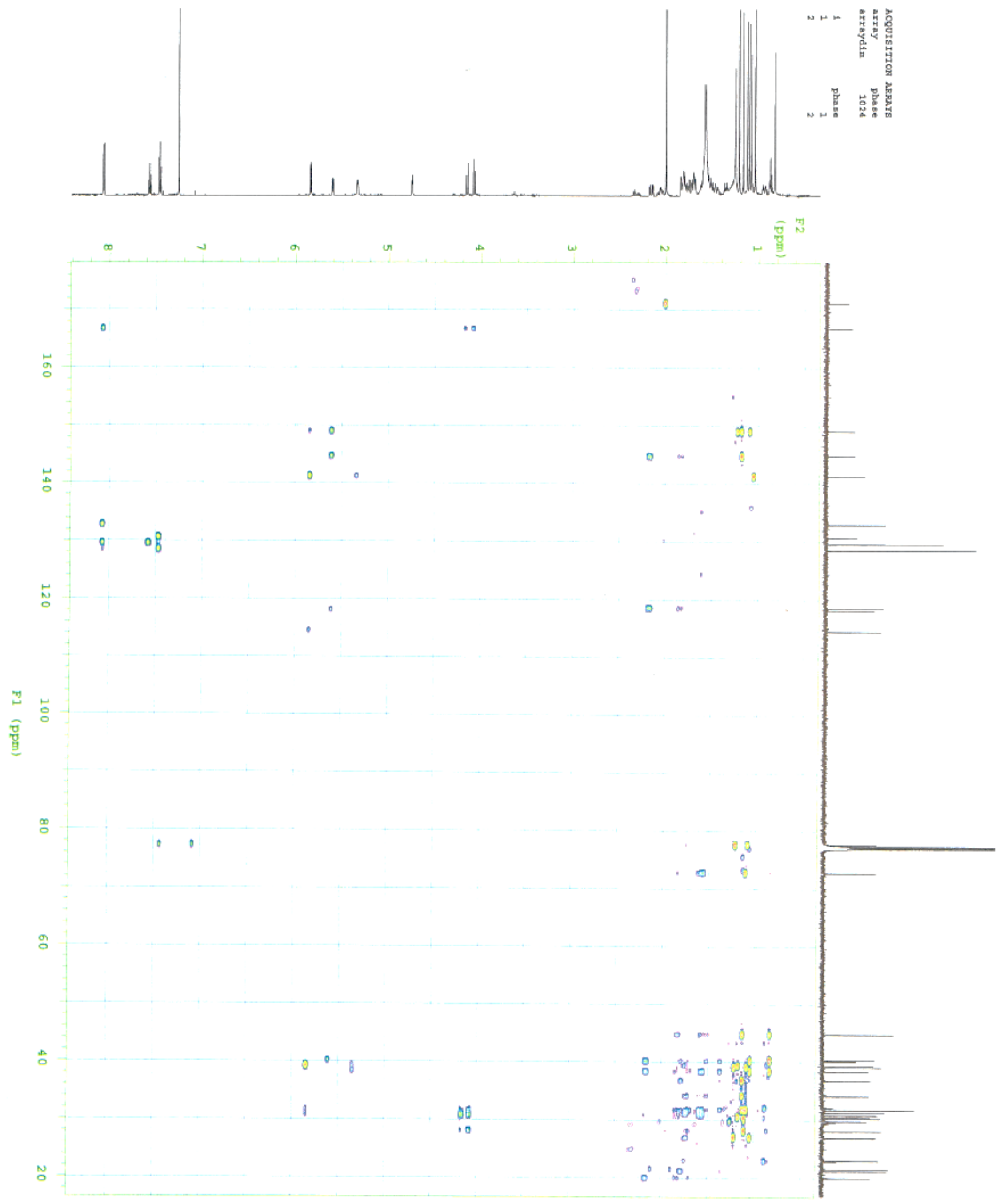

S20. HMBC spectrum of **3**.

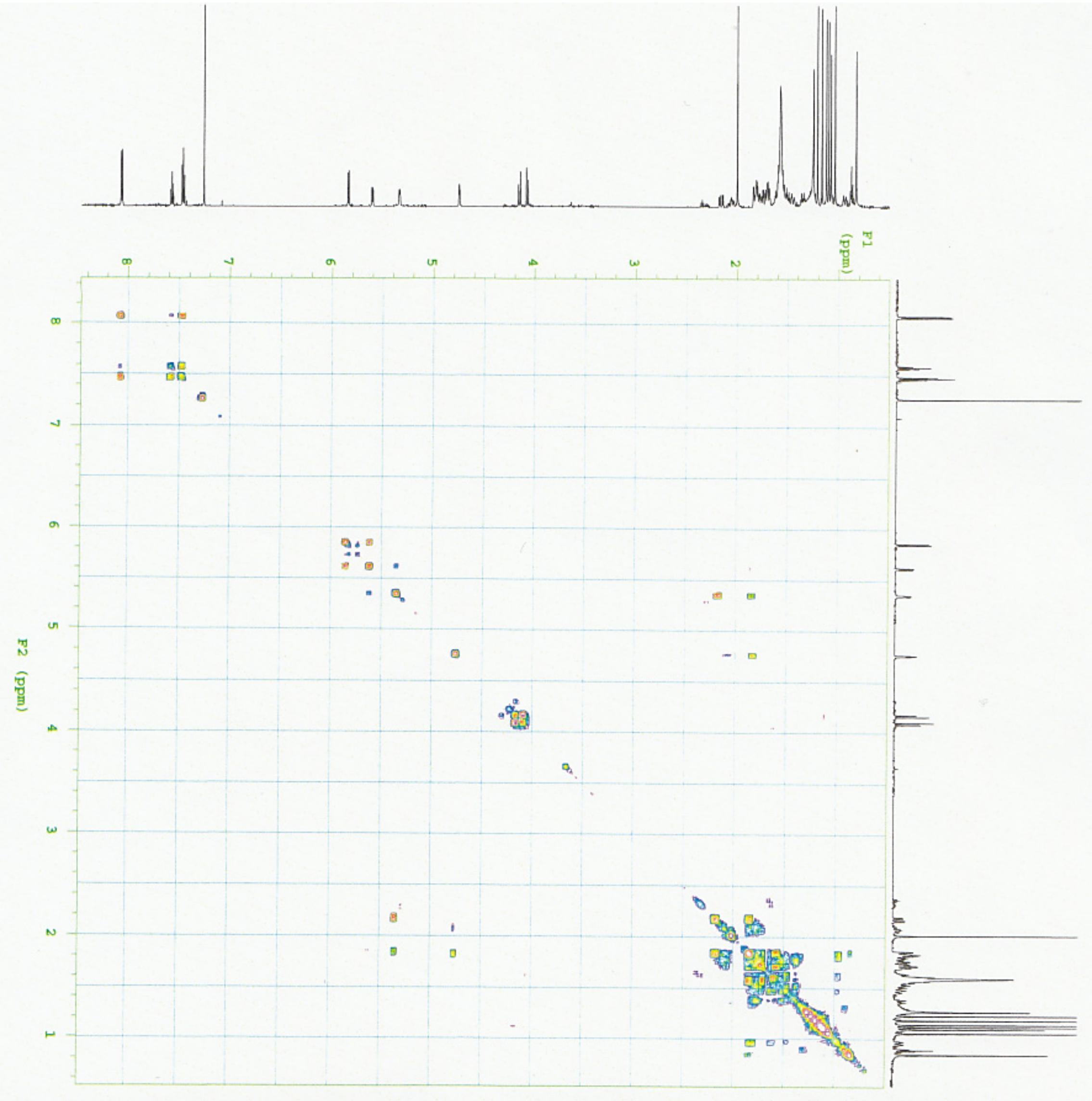

S21. ^{1}H-^{1}H COSY spectrum of **3**.

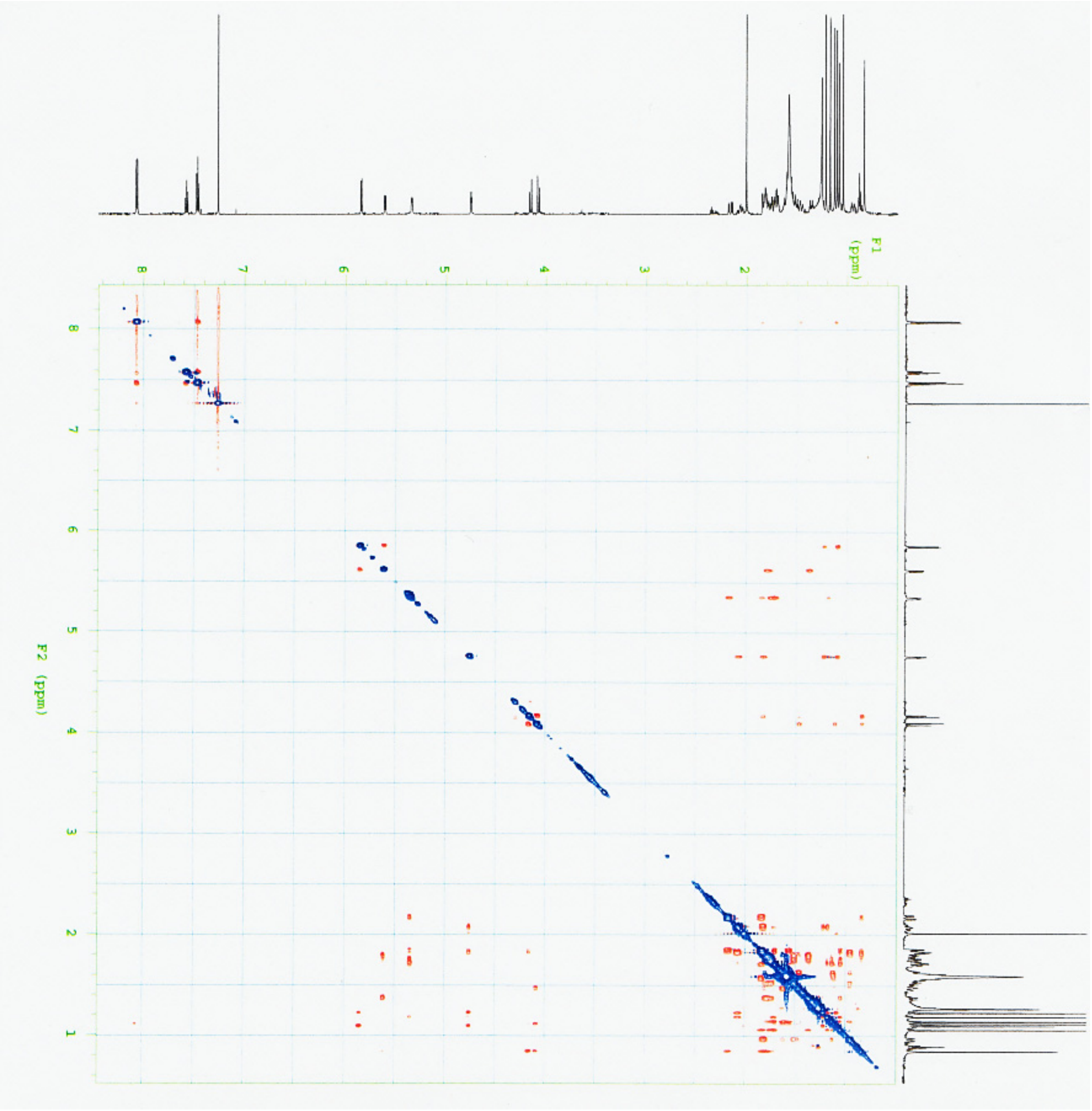

S22. NOESY spectrum of **3**.

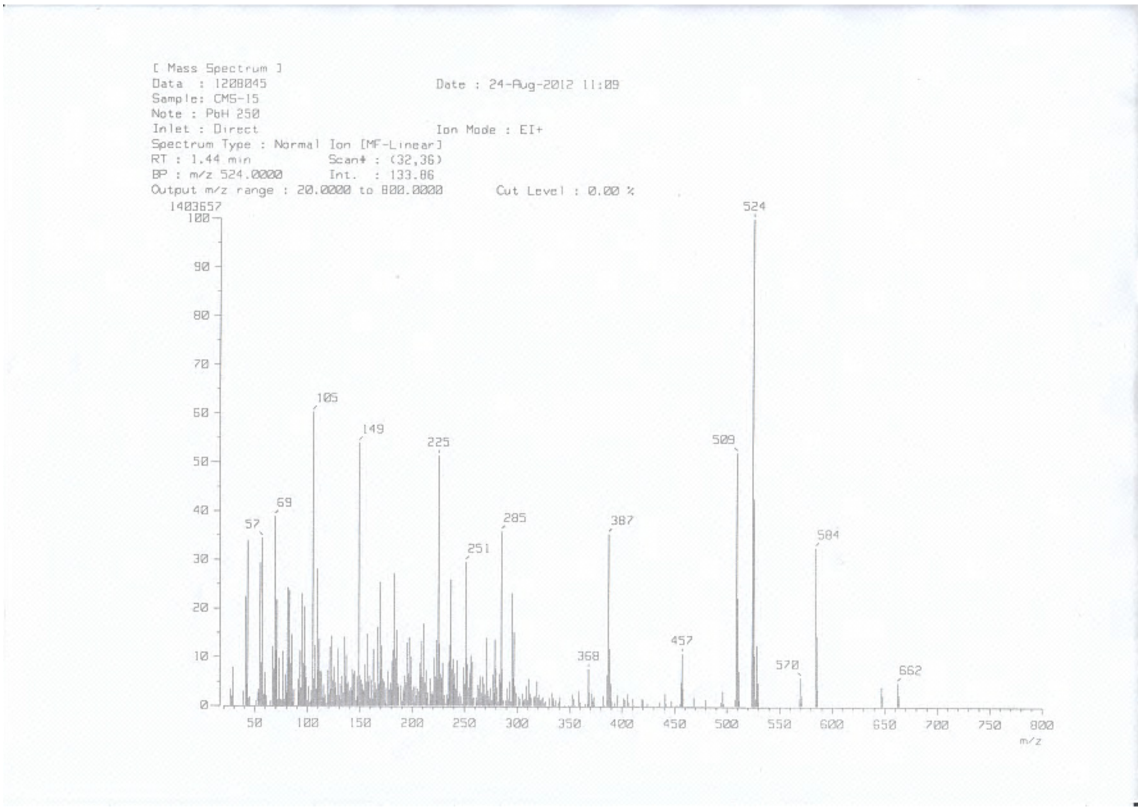

S23. EIMS of **3**.

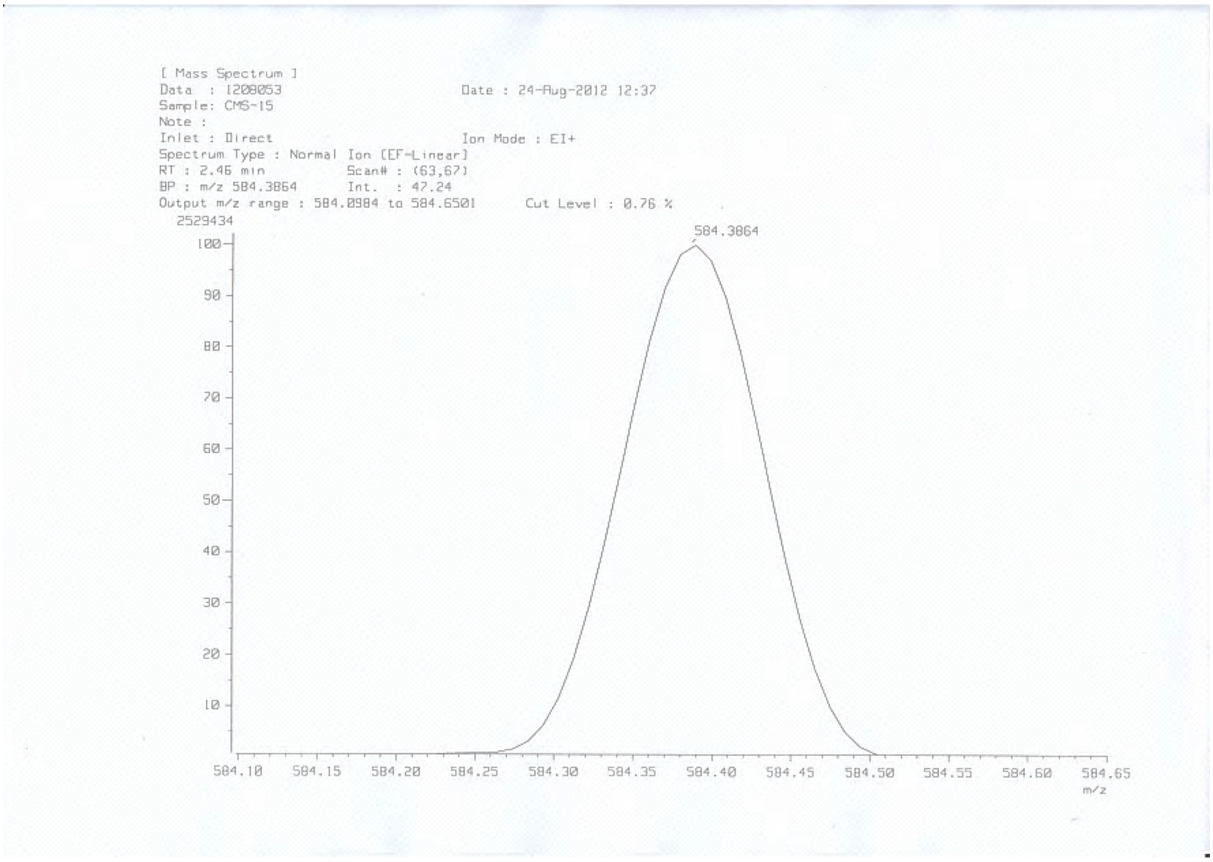

S24. HREIMS of **3**.
